# Chiral cadmium–amine complexes for stimulating non-linear optical activity and photoluminescence in solids based on aurophilic stacks†

**DOI:** 10.1039/d4tc01042f

**Published:** 2024-08-23

**Authors:** Kseniia Boidachenko, Michal Liberka, Junhao Wang, Hiroko Tokoro, Shin-ichi Ohkoshi, Szymon Chorazy

**Affiliations:** a Faculty of Chemistry, Jagiellonian University, Gronostajowa 2 30-387 Krakow Poland simon.chorazy@uj.edu.pl; b Doctoral School of Exact and Natural Sciences, Jagiellonian University, Łojasiewicza 11 30-348 Kraków Poland; c Department of Materials Science, Institute of Pure and Applied Sciences, University of Tsukuba, 1-1-1 Tennodai, Tsukuba Ibaraki 305-8573 Japan; d Department of Chemistry, School of Science, The University of Tokyo, 7-3-1 Hongo, Bunkyo-ku Tokyo 113-0033 Japan

## Abstract

The design of high-performance optical materials can be realized using coordination polymers (CPs) often supported by non-covalent interactions, such as metallophilicity. The challenge is to control two or more optical effects, *e.g.*, non-linear optics (NLO) and photoluminescence (PL). We present a new strategy for the combination of the NLO effect of second-harmonic generation (SHG) and the visible PL achieved by linking dicyanidoaurate(i) ions, which form luminescent metallophilic stacks, with cadmium(ii) complexes bearing chiral amine ligands, used to break the crystal's symmetry. We report a family of NLO- and PL-active materials based on heterometallic Cd(ii)–Au(i) coordination systems incorporating enantiopure propane-1,2-diamine (pda) ligands (1-*S*, 1-*R*), their racemate (2), and enantiopure *trans*-cyclopentane-1,2-diamine (cpda) ligands (3-*S*, 3-*R*). Due to acentric space groups, they exhibit the SHG signal, tunable within the range of 11–24% of the KDP reference, which was correlated with the dipole moments of Cd(ii) units. They show efficient blue PL whose energy and quantum yield, the latter ranging from 0.40 to 0.83, are controlled by Cd(ii) complexes affecting the Au–Au distances and vibrational modes. We prove that chiral Cd(ii)–amine complexes play the role of molecular agents for the stimulation of both the NLO and PL of the materials based on aurophilic stacks.

## Abstract 2

Cd(ii) complexes with chiral amines play a dual role in materials incorporating aurophilic stacks, first, in breaking the symmetry to generate NLO activity, and second, in governing the Au⋯Au distances to give tunable charge transfer luminescence.

## Graphical abstract

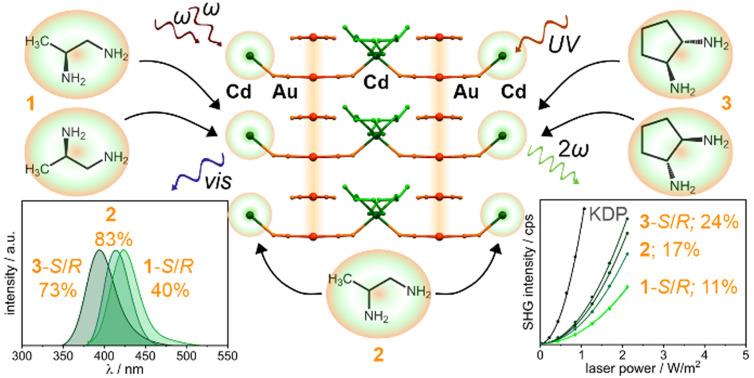

## Introduction

Considerable scientific interest in coordination polymers (CPs), including metal–organic frameworks (MOFs), is related to their high crystallinity, porosity, high modularity, and diverse functionality.^1–3^ They demonstrate a broad range of properties, including sorption, catalytic, electrical, magnetic, and optical ones.^2–8^ New CPs can be constructed by reticular synthesis, *i.e.*, the process of assembling pre-designed molecular building blocks, which offers control over the properties and stability of a target system.^9–15^ Among others, CPs are efficient solid lumiphores displaying a wide range of luminescent effects resulting from their multifaceted nature.^7,16–20^ They show photoluminescence (PL) related to electronic transitions within metal ions, organic ligands, or guest molecules, as well as resulting from the interaction between metal ions and ligands,^17^ becoming applicable in displays, light-emitting diodes (LEDs), optical communication, photovoltaics, chemical sensors, thermometry, and bioimaging.^21–25^

Among other optical materials, the design of chiral systems exploring enantiopure molecular building blocks was intensively addressed.^26–28^ Undoubtedly, CPs or, more generally coordination compounds, offer a convenient opportunity for the rational design of chiral materials due to the broad access to chiral organic ligands and the resulting chiral metal complexes.^29–32^ In general, materials of non-centrosymmetric crystal structures, including chiral ones, are of special interest owing to their distinct physical properties, *e.g.*, ferroelectricity, piezoelectricity, and second-harmonic generation (SHG), which are useful in information storage, electro-optical and nonlinear optical (NLO) devices, light modulators, asymmetric catalysis, chiral separation, *etc.*^33–40^ One of the most important NLO effects is the SHG, in which a material mediates the “adding-up“ of two photons to form a new one with twice the frequency, which is widely used in the laser industry, optoelectronic technologies, and optical microscopy in biological and medical applications.^33,41–44^ Chiral CPs have attracted a lot of attention towards the SHG activity due to their high enantiomeric purity, as well as high structural modularity, which allows obtaining analogous structures enabling the study of the influence of various factors on NLO in crystalline solids.^29–32,45–60^

Three main strategies were employed in the construction of non-centrosymmetric CPs.^29–32^ The first involves the spontaneous generation of chirality from achiral building blocks in the self-assembly process.^49–52^ Although this is desirable because chiral building blocks are more expensive and require often complex syntheses, this process is not fully understood and is hard to predict. We, for instance, showed that this strategy can be supported by playing with rich supramolecular interactions involving cyanido transition metal complexes or employing sterically expanded ligands.^50,51^ In the second method, chiral CPs can be synthesized by metal salts and achiral ligands under the chiral agent influence;^53^ however, the third, most effective, approach still assumes to use the enantiopure ligands or counter-ions, thus it is worth exploring easily accessible chiral species.^54–60^

In these regards, aiming at multifunctional optical materials, research efforts could be directed toward the generation of both NLO and PL in a single-phase material that might be realized by exploiting chiral molecular building blocks.^32,50,60^ To achieve this, we focused our attention on luminescent gold(i) cyanido complexes, *i.e.*, [Au^I^(CN)_2_]^−^ ions. Gold(i) complexes have been known for a long time but they are still of great interest due to their applications, *e.g.*, as semiconductors, or in medicine.^61–63^ The [Au^I^(CN)_2_]^−^ ions can serve as molecular bridges for heterometallic CPs;^64^ however they arouse special attention due to their unique optical properties.^65^ Some metal ions with the *n*d^10^ valence electron configuration interact with each other despite different chemical environments.^65,66^ These interactions are named metallophilic, *e.g.*, aurophilic in the case of Au(i) complexes, and cause the intermetallic distance to be shorter than the sum of van der Waals radii.^65^ The d^10^⋯d^10^ closed-shell aurophilic interactions can be observed in dicyanidometallate-based systems, because of the small effect of steric hindrance on metal centers, which sometimes obscures the extent of these interactions. The remarkable feature is that the Au(i)⋯Au(i) pair reveals the PL under the irradiation of UV light due to the related charge transfer electronic transition. Thus, several studies were reported on the development of light-emitting materials based on [Au^I^(CN)_2_]^−^ complexes.^67–78^ Particular attention is paid to the tuning of the emission maximum of gold(i)-based systems as a function of interatomic distance that can be modified by steric effects of applied building blocks or by external stimuli.^68,79,80^ We have undertaken the challenge to not only prepare novel solid luminophores based on CPs incorporating emissive aurophilic aggregates but also introduce the NLO function to them to achieve a new class of optical materials supported by metallophilicity. We selected Cd^2+^ ions as the accompanying second metal centers as they also reveal the d^10^ valence configuration eliminating emission quenching through low-lying excited states.^81,82^ There has been a limited number of works on the Au(i)–M(ii) (d^10^) systems.^67–72^ To ensure the noncentrosymmetric organization of crystal structures, we decided to use chiral aliphatic 1,2-diamines, propane-1,2-diamine (pda), and *trans*-cyclopentane-1,2-diamine (cpda), having the *N*,*N*-bidentate character suitable for the coordination to Cd(ii) centers, working then as chirality-bearing molecular agents.^67–72^ Exploring the combination of these rationally selected molecular building blocks, we report the syntheses, structures, and optical properties of a family of crystalline solids, {Cd^II^(*S*/*R*-pda)_2_[Au^I^(CN)_2_]}[Au^I^(CN)_2_] (*S*-pda, 1-*S*; *R*-pda, 1-*R*), {Cd^II^(pda)_2_[Au^I^(CN)_2_]_2_} (2), and {Cd^II^(*S*/*R*-cpda)_2_[Au^I^(CN)_2_]}[Au^I^(CN)_2_] (*S*-cpda, 3-*S*; *R*-cpda, 3-*R*), all incorporating infinite aurophilic stacks together with coordination parts. These air- and well-thermally stable materials exhibit the conjunction of SHG activity and intense blue PL, both properties efficiently stimulated by chiral Cd(ii)–amine complexes as was discussed based on the results of thorough experimental studies.

## Results and discussion

### Structural studies

**Fig. 1:**
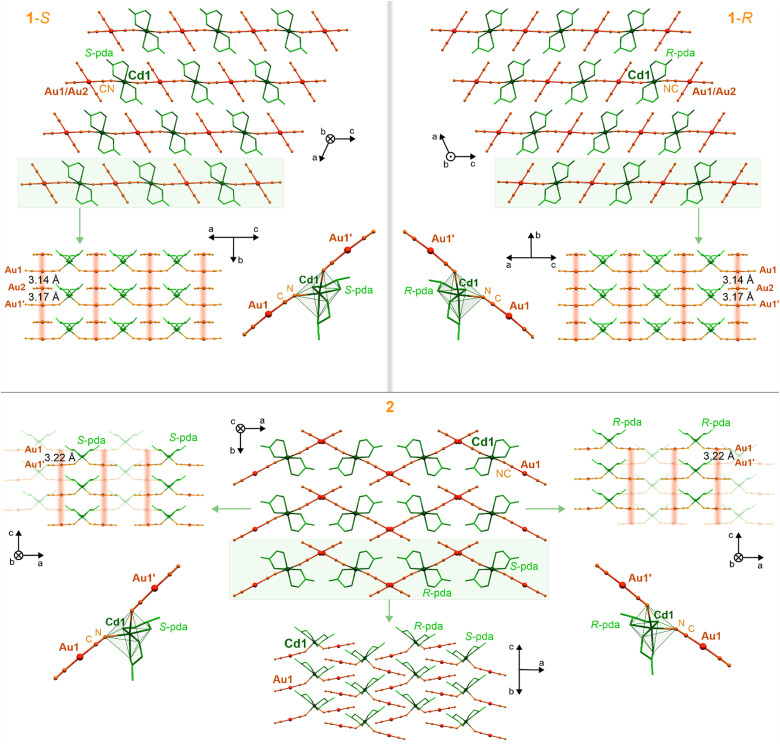
The representative views of the crystal structures of 1-*S* (top left panel), 1*-R* (top right panel), and 2 (bottom), including the crystal packing along the polar crystallographic axes, visualization of the Au⋯Au metallophilic interactions pattern with depicted inter-gold distances, and demonstration of the Cd(ii) coordination sphere together with attached dicyanidoaurate(i) ions. The aurophilic interactions were indicated by a reddish background.

**Fig. 2:**
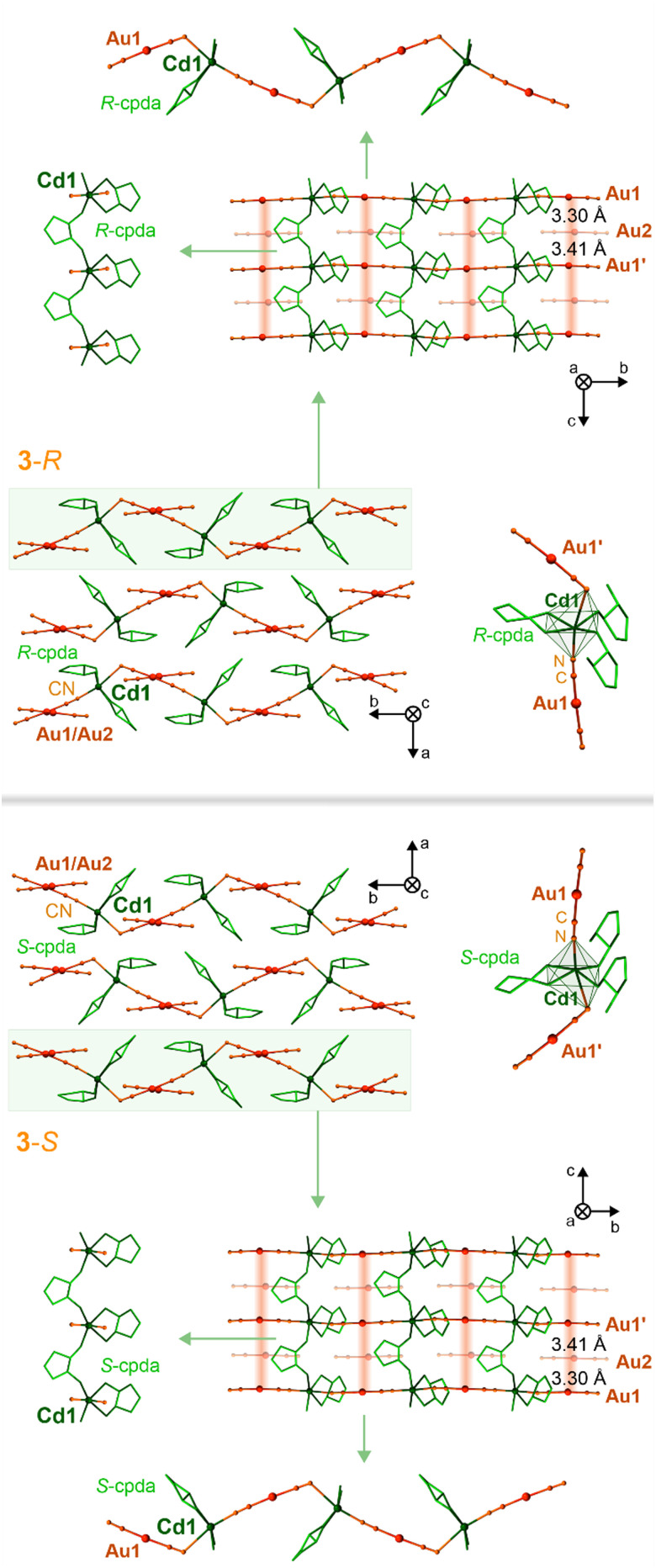
The representative views of the structures of 3-*S* (top), and 3-*R* (bottom), including the crystal packing along the polar crystallographic axes, the coordination spheres of Cd(ii) centers together with attached dicyanidoaurate(i) ions, the single hybrid coordination layer based on inorganic Cd–Au and organic {Cd–(cpda)–Cd} linkages, shown together with supported aurophilic interactions, and the insights into intralayer cyanido-bridged and cpda-bridged chains. The aurophilic interactions were indicated by a reddish background.

Single crystals of 1-*S*, 1-*R*, 2, 3-*S*, and 3-*R* were obtained from the aqueous-methanolic solutions containing Cd^2+^, [Au^I^(CN)_2_]^−^, and the appropriate form of a chiral ligand, *i.e.*, (*S*)-(−)-propane-1,2-diamine (*S*-pda) (1-*S*), (*R*)-(+)-propane-1,2-diamine (*R*-pda) (1-*R*), propane-1,2-diamine (racemic mixture, pda) (2), (1*S*,2*S*)-*trans*-cyclopentane-1,2-diamine (S-cpda) (3-*S*), and (1*R*,2*R*)-*trans*-cyclopentane-1,2-diamine (*R*-cpda) (3-*R*) (Fig. S1, ESI†). The obtained materials were first characterized using CHN elemental analysis, IR absorption spectroscopy, and thermo-gravimetry (TG) (Fig. S2 and S3, and Experimental section in the ESI†), and further by X-ray diffraction (XRD) methods (Fig. 1, 2 and Fig. S4–S12, Tables S1–S8, ESI†). The single-crystal XRD experiments indicate that enantiopure crystals of 1-*S* and 1-*R* crystallize in a monoclinic *C*2 space group (Table S1, ESI†). They are composed of cationic cyanido-bridged {Cd^II^(*S*-pda)_2_[Au^I^(CN)_2_]}_*n*_^*n*+^ (1-*S*) or {Cd^II^(*R*-pda)_2_[Au^I^(CN)_2_]}_*n*_^*n*+^ (1-*R*) chains, accompanied by [Au^I^(CN)_2_]^−^ counter-ions (Fig. 1 and Fig. S4–S6, ESI†). These coordination polymers are based on Cd(ii) centers bearing two diamine ligands each, which are further placed between two [Au^I^(CN)_2_]^−^ ions connected through cyanido bridges. This gives octahedral Λ-*cis*-[Cd^II^(*μ*-NC)_2_(*S*-pda)_2_] and Δ-*cis*-[Cd^II^(*μ*-NC)_2_(*R*-pda)_2_] complexes in 1-*S* and 1-*R*, respectively (Fig. S6 and Table S7, ESI†). The heterometallic {Cd^II^–Au^I^}_*n*_ chains, decorated with chiral diamine ligands, are connected with non-coordinated [Au^I^(CN)_2_]^−^ ions by Au⋯Au interactions providing supramolecular layers (Fig. 1 and Fig. S6, Table S8, ESI†). Aurophilic interactions are depicted by the short Au–Au distances of 3.14 and 3.17 Å, identical in both enantiomorphs (Table S4, ESI†).^65^ These interactions extend along the polar crystallographic *b* axis, and the Au(chain)⋯Au(ion)⋯Au(chain) sequence leads to linear metallic stacks composed of coordinated and uncoordinated cyanidometallates. These chains are crossed perpendicularly by cyanido-bridged {Cd^II^–Au^I^}_*n*_ CPs, giving the mentioned layered structure. They are spaced by the intermetallic distance of *ca.* 7.5 Å and bonded by the N–H⋯NC hydrogen bonds, engaging terminal cyanido ligands of non-coordinated [Au^I^(CN)_2_]^−^ ions and the NH_2_ groups of pda ligands (Table S4, ESI†). The amine groups form also hydrogen bonds within the coordination-metallophilic layers interacting with CN^−^ ligands, as well as participating in Au⋯H–N interactions.^83^ As the enantiopure ligands are placed in isostructural 1-*S* and 1-*R*, their chiral structures are perfect mirror images (Fig. 1 and Fig. S4–S6, ESI†). Thus, they can be considered as two enantiomorphic compounds which were further confirmed by circular dichroism (CD) spectra that contain the distinct complex bands in the UV range being the mirror images for 1-*S* and 1-*R* (Fig. S17, ESI†).

Materials 1-*S* and 1-*R* are obtained from hydrochlorides of enantiopure *S*-pda and *R*-pda, respectively (Fig. S1, ESI†). When the mixture of hydrochlorides of *S*-pda and *R*-pda ligands is used, the decisive influence on the product formation is the ionic strength of the solution. In the presence of a strong electrolyte, the equimolar mixture of 1-*S* and 1-*R* is formed. The removal of an additional electrolyte from the solution inhibits the formation of this mixture and causes the crystallization of a new material, 2, containing both forms of optically pure ligands (Fig. 1 and Fig. S1, ESI†). This system can also be obtained using the electroneutral racemic mixture of pda ligands. Thus, block-shaped crystals of 2 were formed from the aqueous-methanolic solution devoid of additional electrolytes. Despite the presence of both *S*-pda and *R*-pda, 2 crystallizes in the non-centrosymmetric *A*ba2 space group (Table S2, ESI†). Its structure consists of trinuclear {Cd^II^(pda)_2_[Au^I^(CN)_2_]_2_} molecules (not a coordination polymer as 1-*S*/1-*R*), built of two linear dicyanidoaurate(i) ions bridged by single cyanido ligands to the Cd^II^ center, which is coordinated also by four N-atoms from two bidentate pda ligands (Fig. 1 and Fig. S7, S8, Tables S5, S7, S8, ESI†). Compound 2 contains equal proportions of *S*-pda and *R*-pda ligands, which are related symmetrically by glide planes; thus, they form achiral frameworks as confirmed by the lack of the signal in the expected UV range of the CD spectrum (Fig. S17, ESI†). The resulting octahedral Cd^II^ complexes, always containing a single type of the pda enantiomer, Λ-*cis*-[Cd^II^(*μ*-NC)_2_(*S*-pda)_2_] or Δ-*cis*-[Cd^II^(*μ*-NC)2(*R*-pda)_2_], extend alternately along the a axis (Fig. 1 and Fig. S8, ESI†). The {Cd^II^Au^I^_2_} molecules link each other through aurophilic interactions which are depicted by the Au⋯Au distances of 3.22 Å and create non-perfectly linear metallophilic stacks along the c axis. This further results in undulating supramolecular layers based on aurophilic and coordination parts. The whole structure, within and between these layers, is stabilized by a rich hydrogen bonding network, involving terminal CN^−^ ligands and H-atoms from coordinated amine groups (Table S5, ESI†).

By replacing *S*-pda and *R*-pda ligands with more expanded (1*S*,2*S*)-*trans*-cyclopentane-1,2-diamine (*S*-cpda) and (1*R*,2*R*)-*trans*-cyclopentane-1,2-diamine (*R*-cpda), layered Cd^II^–Au^I^ coordination polymers of 3-*S* and 3-*R*, respectively, were obtained (Fig. 2 and Fig. S1, S2, S9–S11, Tables S3 and S6–S8, ESI†). Both materials crystallize in the orthorhombic *P*2_1_2_1_2 space group with one Cd^2+^ ion, two [Au^I^(CN)_2_]^−^ complexes, and two cpda ligands in the asymmetric unit (Fig. S11, ESI†). Their structure is composed of {Cd^II^(*S*-cpda)}^2+^ and {Cd^II^(*R*-cpda)}^2+^ fragments in 3-*S* and 3-*R*, respectively, connected by *trans*-positioned dicyanidoaurate(i) metalloligands into zig-zag {Cd^II^–Au^I^}_*n*_ chains. They are further combined by bridging cpda molecules forming {Cd–(cpda)–Cd} linkages into hybrid I^1^O^1^ coordination layers (Fig. 1, ESI†).^84^ The rectangular spaces within these layers are filled with non-coordinated [Au^I^(CN)_2_]^−^ ions. They participate in the formation of infinite aurophilic stacks along the *c* axis with Au⋯Au distances of 3.30 and 3.41 Å (Fig. 2 and Fig. S11, Table S6, ESI†). These metallophilic chains involve the [Au^I^(CN)_2_]^−^ ions from the hybrid layers which contribute to the overall stabilization of the materials. The interlayer interactions are realized by a rich network of hydrogen bonds (Table S6, ESI†). Overall, the chiral structures of 3-*S* and 3-*R* are perfect mirror images; thus, these two compounds are enantiomers as also confirmed by the distinct signals in the UV range of the respective CD spectra. As expected, these CD bands are mirror images for 3-*S* and 3-*R* (Fig. S17, ESI†). A distinct difference between pda-based 1-*S*, 1-*R*, and 2, and cpda-based 3-*S* and 3-*R* is how [Au^I^(CN)_2_]^−^ ions are coordinated to Cd^2+^ ions. In the first group, there is a single bent molecular bridge with the Cd–N–C angle of *ca.* 142° (Fig. S6, S8 and Tables S4, S5, ESI†). In 3-*S* and 3-*R*, there are two types of cyanido bridges, one linear and one bent with the Cd–N–C angles of 175° and 118°, respectively (Fig. S11 and Table S6, ESI†). With increasing a bridge angle, the distance between the CN^−^ group and Cd(ii) centers decreases, proving the strengthening of this bond. This feature is reflected in the IR spectra, where rich absorption related to the C≡N stretching vibrations is observed (Fig. S2, ESI†).^50–53,85–87^

**Fig. 3:**
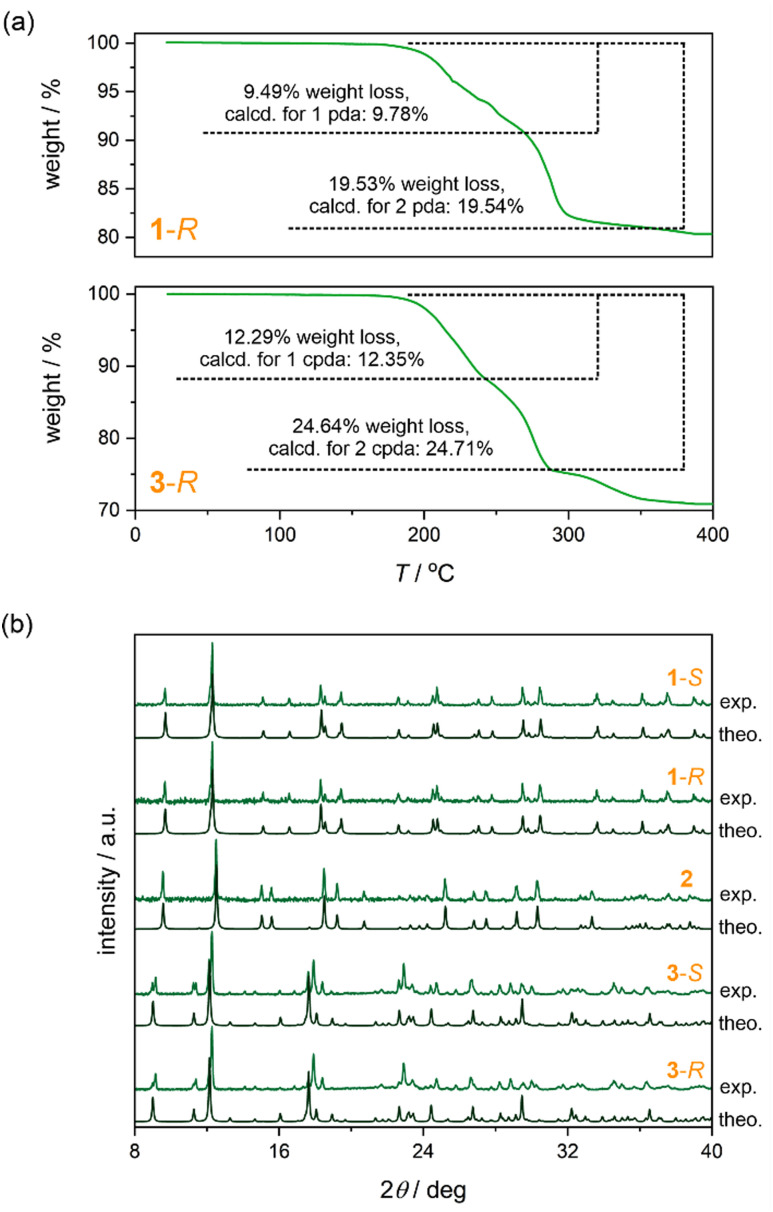
The air- and thermal stability as well as phase purity of obtained materials: (a) the TG curves of 1-*R* and 3-*R*, and (b) the comparison of experimental (*T* = 300 K) powder X-ray diffraction patterns of 1-*S*, 1-*R*, 2, 3-*S*, and 3-*R* with the patterns calculated from the respective structural models obtained from the SC-XRD structural analysis (*T* = 100 K).

Upon heating, the structures of 1-*S*, 1-*R*, 2, 3-*S*, and 3-*S*, exhibit a thermal expansion associated with the increase in bond lengths resulting from *T*-activated vibrational modes (Tables S1–S3, ESI†).^87^ All materials exhibit a progressive heating-induced elongation of the C and N thermal ellipsoids along the axis perpendicular to cyanido bridges which results in the contraction of the lattice parameter along directions where also [Au^I^(CN)_2_]^−^ complexes are placed (Fig. 1, 2 and Tables S4–S6, ESI†). As a result, only a slight change in bending the coordination parts is observed. Simultaneously, upon heating, the Au⋯Au distances increase by *ca.* 2% (Tables S4–S6, ESI†), which is crucial for the PL of obtained solids (see below). Nevertheless, the thermal structural variation is small which, together with the lack of solvent molecules and the substantial combination of coordination skeletons with metallophilic stacks and the rich hydrogen bonding systems, result in the perfect stability of the materials in the air as well as the good thermal stability up to at least 200 °C as depicted by the TG curves (Fig. 3a and Fig. S3, ESI†). Heating of the materials to higher temperatures leads to the removal of organic ligands and the subsequent decomposition. The structural models obtained from the SC-XRD analyses are valid for the powder samples used in optical studies as proven by P-XRD (Fig. 3b and Fig. S12, ESI†).

### Second-harmonic generation activity

**Fig. 4:**
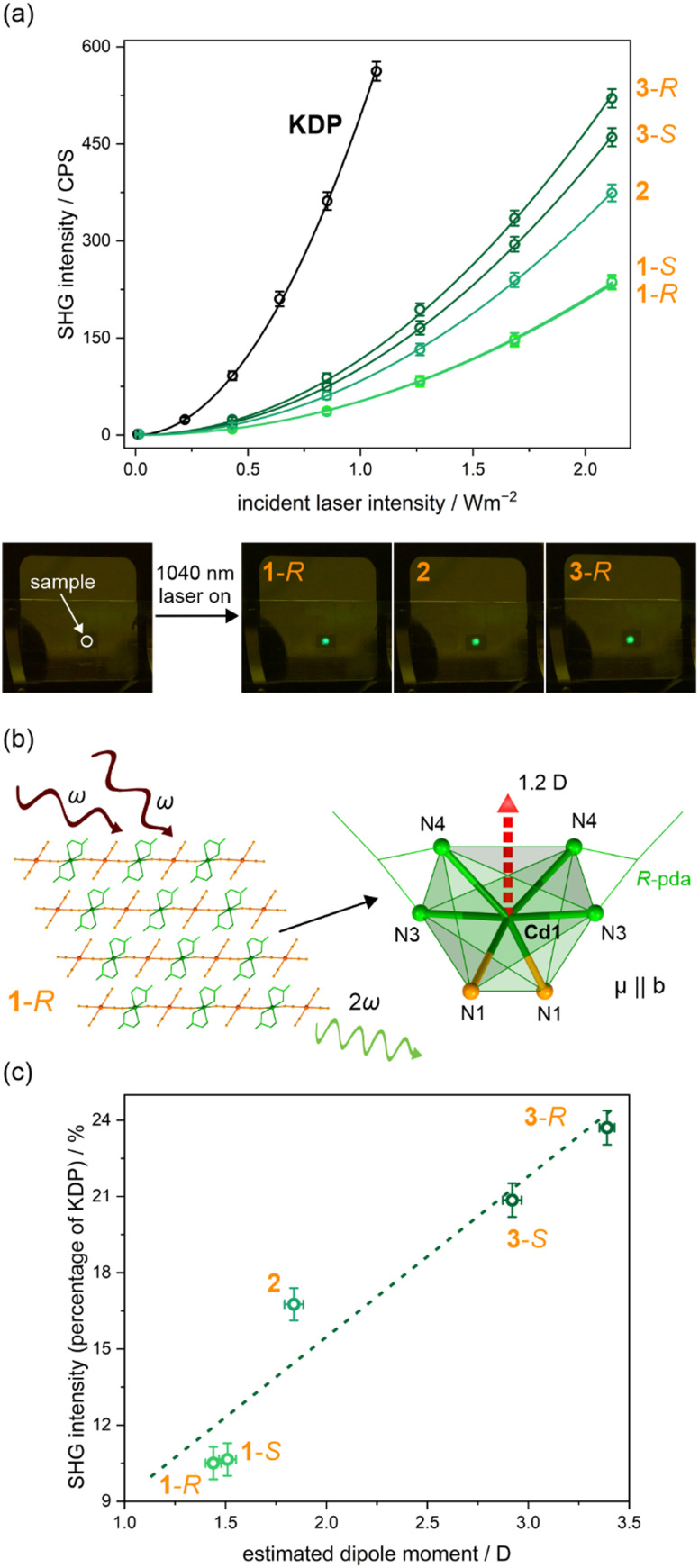
The room-temperature SHG properties of 1-*S*, 1-*R*, 2, 3-*S*, and 3-*S* compared with a KDP reference sample: (a) the SHG intensity in the function of excitation intensity with the photos of observed SHG light under the 1040 nm laser irradiation, (b) the schematic illustration of the SHG effect and the direction of dipole moment vector (red arrow) in a the {CdN_6_} distorted octahedral Cd(ii) complexes in 1-*R*, and (c) the dependence of SHG intensities on the estimated dipole moment calculated for the incorporated {CdAu_2_} units in obtained materials. In (a), colored circle points represent the experimental data while solid lines show the best fits following the quadratic function.

As all the presented compounds crystallize in acentric space groups (Tables S1–S3, ESI†), we investigated the second-harmonic generation (SHG) property to evaluate their potential as NLO materials. The measurements were carried out on polycrystalline samples (100–150 μm of the average diameter of crystals) using a 1040 nm femtosecond pulse laser as incident fundamental light (Fig. 4 and Fig. S13, S14, ESI†).^50^ The resulting light of *ca.* 520 nm was proportional to the square of the excitation light intensity proving a two-photon process. The gathered signals were compared with the potassium dihydrogen phosphate (KDP) reference (Fig. 4a and Table S9, ESI†). Based on this comparison, chiral 1-*S* and 1-*R* materials containing enantiopure pda ligands were found to exhibit SHG efficiencies of little more than *ca.* 10% of KDP reference, while, interestingly a better signal (*ca.* 16% of KDP) was observed for acentric 2 material incorporating both pda enantiomers. The best SHG efficiencies with intensity values over 20% of KDP were found for bulk samples of 3-*S* and 3-*R*.

To better understand the differences in the SHG intensity, one can consider the local dipole moment and the dipole–dipole interactions in the structures of the presented materials.^88–93^ For this purpose, a simple bond-valence approach was to calculate the direction and magnitude of the dipole moment of complexes of all crystallographically independent ions, taking into account their non-ideal polyhedra from XRD experiments at 300(2) K. The Debye equation, *μ* = *n*·*e*·*R*, where *μ* is a net dipole moment, *n* is a total number of electrons, *e* is a charge on an electron, and *R* is a difference between “positive” and “negative” charge, was used to calculate the dipole moment of individual Cd–N and Au–C bonds. The distribution of electrons on Cd, N, Au, and C atoms, was estimated using bond-valence theory, *i.e.*, *S*_i_ = exp[(*R*_0_–*R*_i_)/*B*], where *R*_0_ and *B* are empirical constants, *R*_i_ is s bond length.^94,95^ The results and detailed information of these calculations are described in the ESI† (Fig. S15, S16 and Tables S10, S11, and the related comment in the ESI†). Generally, in the example of the Cd(ii) coordination environment in 1-*R*, {CdN_6_} (Fig. 4b), the atomic valences were calculated to be Cd(+1.93), N1(NC) (−0.26), N3(amine) (−0.39), and N4(amine) (−0.32), to give dipole moments of 5.6, 6.4, and 6.0 D (Debye) for Cd1–N1, Cd1–N3, and Cd1–N4, respectively (Table S10, ESI†). The vector sum of dipole moments for all six Cd–N bonds gives the net dipole moment of 1.1 D, directed along the b axis towards the plane formed by four coordinated nitrogen atoms of pda amine ligands (Fig. S15 and Table S10, ESI†). The calculations for similar Cd(ii) complexes in 1-*R* and 2 give the vector sum of dipole moments directed toward the *b* and *c* directions, respectively, with a similar magnitude of 1.2 D (Fig. 4b and Fig. S15, Table S10, ESI†). For Cd(ii) complexes in 3-*S* and 3-*R*, the calculated dipole moment vector summation gives the net dipole moment of *ca.* 2.5 D, larger than for Cd(ii) complexes in 1-*S*, 1-*R*, and 2, directed roughly along the Cd1–N3 bond axis towards the bent cyanido bridge (Fig. S15 and Table S10, ESI†). The polyhedra of [Au^I^(CN)_2_]^−^ complexes were also calculated. In 1-*S*, 1-*R*, and 2, the magnitude of the vector sum of dipole moments is *ca.* 0.3 D for bridging complexes, while for non-coordinated ones it is less than 0.07 D. Respective calculations in 3-*S* and 3-*R* give values of 0.41–0.54 D. These results reflect the distortion from ideal polyhedra of Cd(ii) and Au(i) complexes, as shown by Continuous Shape Measure (CShM) analysis (Fig. S16, and Tables S7, S8, ESI†).^96^ The Cd(ii) complexes in all compounds, 1-*S*, 1-*R*, 2, 3-*S*, and 3-*R*, can be described by octahedrons but with high CShM values (2.2–2.8), which proves their significant deformation. The highest values were calculated for Cd(ii) complexes in 3-*S* and 3-*R*, which are also described by the highest magnitudes of the net dipole moment (Tables S7 and S10, ESI†). Similarly, the higher CShM values (linear geometry) for coordinated Au(i) complexes reflect the higher dipole moment of these complexes (Tables S8 and S11, ESI†). However, it should be noted here that in all cases the dipole moments related to the Au(i) complexes are much smaller than those for Cd(ii) complexes. This is related to the intrinsically chiral character of the latter complexes and the closely linear shape of the Au(i) complexes. The applied method of estimating the dipole moments is error-prone as it does not take into account the precise distribution of electrons in complexes, which is affected by ionic interactions and the formation of hydrogen bonds involving cyanido ligands and N–H amine groups,^89,94^ however, it reflects main trends.

All the above-considered dipole moments of Cd(ii) and Au(i) complexes do not fully contribute to the net dipole moment without any cancellation. The polar two-fold axis of rotation is oriented parallel to the *b* direction in 1-*S* and 1-*R*, and along the *c* direction in 2, so the components of the net dipole moment of adjacent complexes in perpendicular directions will cancel out (Fig. 1 and Fig. S5, S7, ESI†). Hence, there is no net perpendicular polarization, and only the parallel component will contribute to the resultant dipole moment. Full crystal packings of the Cd(ii) and Au(i) complexes in 1-*S*/1-*R* and 2, labeled with the resulting not-canceled net dipole moment are presented in Fig. S15 (ESI†). In 1-*S* and 1-*R*, favorable dipole–dipole interactions occur between each Cd(ii) complex belonging to coordination chains, and also between Cd(ii) and coordinated Au(i) complexes, while the contribution of non-coordinated complexes is rather negligible (Table S11, ESI†). Moreover, the offset of a 1/2 *b* period between adjacent chains additionally favors the interactions between those dipoles. A similar interaction scheme can be observed in the crystal structure of 2, for which favorable dipole–dipole interactions occur between each metal complex, between and within the supramolecular layers. The materials 3-*S* and 3-*R* crystallize in a non-polar *P*2_1_2_1_2 space group which causes the resulting dipole moment in the structure is not oriented along the polar axis as in 1-*S*, 1-*R*, and 2, but neighboring complexes contribute to the net dipole moment with cancellation (Fig. S10, ESI†). However, the local dipole interactions within the symmetrically independent {CdAu_2_} moieties are favorable. According to the anionic group theory, the overall NLO effects reflect the microstructure of relevant ionic groups.^97,98^ Therefore, if the local dipole moment of {CdAu_2_} fragments is comparable, it should represent the NLO effects quite well. In 1-*S* and 1-*R*, the local dipole moment is the sum of the moments of one Cd(ii) complex and coordinated Au(i) metalloligand, while the participation of non-bridging [Au^I^(CN)_2_]^−^ counterions is negligible (Table S11, ESI†). In 2, the positive interactions of dipole moments of the Cd(ii) complex and two Au(i) metalloligands give a higher local dipole moment, which results in better SHG efficiency (Table S9, ESI†). The strongest SHG signal was detected for 3-*S* and 3-*R*, which is caused by the highest local dipole moments of three individual polyhedra in the {CdAu_2_} fragment. It was found that the SHG efficiencies are linearly dependent on the summed dipole moment of {CdAu_2_} moieties in 1-*S*, 1-*R*, 2, 3-*S*, and 3-*R* (Fig. 4c and Fig. S16, ESI†). From this discussion, one can deduce that the SHG efficiency is dependent on the dipole moments of properly aligned Cd–Au fragments among which the more significant role is played by the Cd(ii) complexes providing larger individual contributions. The above analysis, even though it is based on approximations and does not take into account the supramolecular interactions, provides important information that the SHG efficiencies reflect the local microstructure; nevertheless, future studies on single crystals will give more accurate results than presented powder SHG tests.^99,100^

### Photoluminescence and other optical properties

**Fig. 5:**
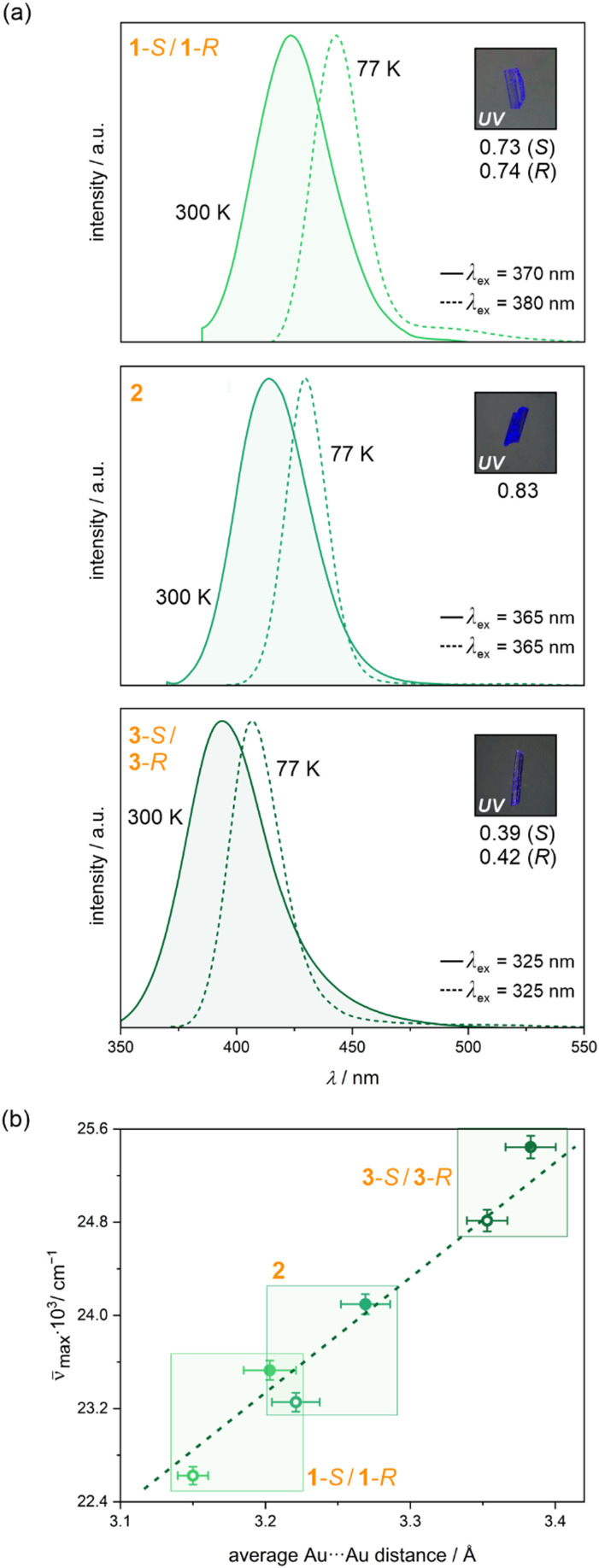
Photoluminescent properties of 1-*S*, 1-*R*, 2, 3-*S*, and 3-*R*: (a) the comparison of emission spectra at 77 and 300 K under the indicated excitation, shown with the photo of the sample under UV light and the values of quantum yields, and (b) the plot of the average Au⋯Au distance in the structure *versus* the wavenumber of the emission band maximum. In (b), the comparison of high-temperature (300 K) emission and structural features are presented as full circles, while empty circles show the low-temperature emission collected at 77 K which was compared with the structural data from 100 K.

Aurophilicity-based materials are found to exhibit efficient photoluminescence, the energy of which is strongly dependent on the interatomic Au–Au distances.^65–80,101–103^ Upon UV excitation related to the broad absorption (Fig. S17, ESI†), the powder samples of 1-*S*, 1-*R*, 2, 3-*S*, and 3-*R*, exhibit blue PL, tuned by structural features and temperature (Fig. 5a and Fig. S18–S20, Tables S12 and S13, ESI†). At 300 K, the emission spectrum of 1-*S* and 1-*R* exhibit a single band centered at 425 nm, while 2 reveals similar emission with a maximum at 430 nm. Their excitation spectra, monitored at emission maximum, are in the lower wavelength range almost the mirror images of emission patterns with a maximum at 370 and 365 nm for 1-*S*/1-*R* and 2, respectively (Fig. S18 and S19, ESI†). Compounds 3-*S* and 3-*R* do not show distinct PL after the 370 nm excitation, however, the use of higher-energy light of 325 nm causes the prominent emission peak at 393 nm (Fig. S20, ESI†). The single emission band in the visible range in all materials is typical for dicyanidoaurate(i)-based systems.^67–78^ Thus, this solid-state PL is associated with the formation of infinite metallophilic {Au–Au}_*n*_ stacks (Fig. 1 and 2). It has been shown that upon photoexcitation the equilibrium Au–Au distance significantly decreases with the transition from the ground to the lowest-energy triplet excited state. Thus, the emissive state formation takes place as a result of a HOMO–LUMO Au-centered excitation transition from an antibonding orbital to a bonding orbital.^104–106^ As this emissive state involves also the neighboring Au(i) complex and, partially, attached ligands, it is usually considered as a charge transfer character (metal-to-metal-to-ligand charge transfer, MMLCT).

At low temperatures, the emission spectra of all compounds retain their character, however, the bands are red-shifted and narrowed (Fig. 5a and Fig. S18–S20, ESI†). The 77 K spectrum of 1-*S* and 1-*R* displays a structureless single-emission feature at 442 nm. A similar, single narrow emission band with the maxima at 430 and 403 nm is observed for 2 and 3-*S*/3-*R*, respectively. The shift of the emission maximum upon cooling results from the compression of the structure, thus decreasing the Au⋯Au distances within aurophilic stacks (Fig. S4–S11 and Tables S1–S6, ESI†). It was demonstrated theoretically and experimentally that the Au⋯Au distances in metallophilic systems are usually inversely proportional to emission energies.^79,80^ Indeed, the high-energy blue PL in presented solids can be associated with the change in the distance between Au(i) centers, but the trend is the opposite, similar to polymorphic forms of Zn^II^-[Au^I^(CN)_2_]^−^ frameworks, or Pb(ii)-dicyanoaurate(i) CPs.^68,69^ At 77 K, the average shorter Au⋯Au distances in 1-*S* and 1-*R* (3.14 and 3.17 Å) result in lower energy emission than the Au⋯Au distances in 2 (3.22 Å) and 3-*S*/3-*R* (3.30 and 3.41 Å). These distances lengthen upon heating, causing the blue shift of emission bands, which is proportional to the bond distance change. A plot of the average Au⋯Au distance *versus* emission energy gives a straight line (Fig. 5b). Previous works on the Au(i)-centered emission, in which the higher-energy PL in crystals that exhibit shorter Au⋯Au interactions, has been related to a more constrained photoinduced rearrangement, that does not allow freedom for a drastic structural change in the two complexes comprising the excimer.^104–106^ In the case of more rigid CPs, a drastic structural change within the photoinduced rearrangement and the transition to the triplet excited state, as well as the formation of Au–Au bond, should be much more difficult even at longer intermetallic distances. Nevertheless, the direct relationship between the emission energy and the average Au⋯Au distances in obtained compounds undoubtedly proves that the metallophilic interactions govern the observed photoluminescence.

It should be emphasized that in all compounds a strong PL signal was recorded (Fig. 5b and Table S12, ESI†). The strongest emission, characterized by the room-temperature quantum yield of *Φ* = 0.83 was observed for 2, while a slightly weaker emission signal was detected in 1-*S* and 1-*R* (*ca.* 0.73 of *Φ*). The lowest *Φ* values of *ca.* 0.40 were obtained for 3-*S* and 3-*R*, still high among M^II^–[Au^I^(CN)_2_]^−^ systems.^67–72^ These differences can be assigned to the larger number of quenching C–H/N–H vibration modes.^107,108^ The interaction with the lattice vibrations is also responsible for the Stokes shift, the highest in 3-*S* and 3-*R*, but it can be related, to some extent, to long Au⋯Au distances. The emission decay profiles of the emission were fitted as monoexponential functions with similar values of *ca.* 550 ns at 300 K and *ca.* 930 ns at 77 K (Fig. S18–S20, ESI†). The obtained lifetime value close to the microsecond level corresponds to triplet excited states of dicyanidoaurate(i) ions.^67–72^ To demonstrate the efficient PL, we calculated the radiative (*k*_r_) and nonradiative (*k*_nr_) decay rate constants using relationships *k*_r_ = *Φ*/*τ* and *Φ* = *k*_r_/(*k*_r_ + *k*_nr_). The *k*_r_ values of 1-*S*, 1-*R*, and 2 fall in the range of 13.6 × 10^5^–14.0 × 10^5^ s^−1^, while the relevant values of 3-*S* and 3-*R* are around 8 × 10^5^ s^−1^, illustrating that the *Φ* and *τ* values are governed by the radiative decay rate. Moreover, more than twice the higher values of a nonradiative decay rate constant (1.1 × 10^5^ s^−1^) in the cpda-containing 3-*S* and 3-*R* indicate a distinct role of quenching vibrational modes in the relaxation of the excited states. This can be correlated with the more expanded character of cpda ligands providing more C–H and N–H quenchers in the vicinity of metallophilic stacks.

It is worth mentioning here that materials 1-*S* and 1-*R*, as well as 3-*S* and 3-*R*, are pairs of enantiomers so they are promising candidates for the observation of chiroptical properties, besides the SHG activity and photoluminescence presented above. This goal of further studies stays beyond the scope of this work; however, we preliminarily tested this perspective by collecting the circular dichroism (CD) spectra in the UV-vis range for mentioned enantiomorphic compounds, as well as for achiral compound 2 for the comparison (Fig. S17, ESI†). While for the latter, as expected, we did not observe any noticeable CD signal in the whole investigated wavelength range, the compounds 1-*S* and 1-*R*, as well as 3-*S* and 3-*R*, exhibit pronounced CD bands. The first of these pairs of materials exhibit the broad complex CD signal ranging from 250 to *ca.* 380 nm which covers well the related absorption bands that are lying in a similar region of the electromagnetic spectrum. As these light absorption bands can be mainly assignable to the CT-type electronic transitions within the dicyanidoaurate(i) ions and their metallophilic stacks,^67–78^ the observation of such CD signal indicates the presence of an efficient chirality transfer from the chiral Cd(ii) complexes to the attached Au(i) complexes. A similar situation is observed for 3-*S* and 3-*R*; however, the CD bands were detected in the narrower range of 250–350 nm. This corresponds well to the light absorption bands which are blue-shifted for these materials in comparison to the 1-*S* and 1-*R* pair. The latter effect is analogous to those observed in the emission spectra and it can be similarly ascribed to the modified Au⋯Au distances within the crucial metallophilic stacks. This leads to the conclusion that chiroptical properties can be also generated and modulated within the investigated family of compounds which is expected to be further explored in the context of possible circularly polarized luminescence (CPL) effect utilizing the CT emission related to the aurophilic stacks and the efficient chirality transfer from chiral Cd(ii) complexes. The crucial role of the metallophilic interaction in enabling the chiroptical effects to happen at the edge of UV and visible ranges of the spectrum, instead of only in the deep UV region where the chiral organic ligands can absorb the energy, can be thus postulated.

## Conclusions

We report a convenient synthetic pathway for preparing and optimizing the characteristics of bifunctional materials linking non-linear optics (NLO), represented by second harmonic generation (SHG), with visible photoluminescence (PL). The presented strategy relies on spontaneously formed aurophilic molecular stacks based on dicyanidoaurate(i) ions that undergo the self-assembly with cadmium(ii) complexes bearing chiral diamine ligands, smaller propane-1,2-diamine (pda) or larger *trans*-cyclopentane-1,2-diamine (cpda). Resulting Cd–Au materials reveal good air- and thermal stability thanks to the co-existence of cyanido and organic molecular bridges, supported by aurophilic interactions. The metallophilicity of embedded Au(i) molecular units ensures the efficient visible PL due to the appearance of emissive MMLCT excited states while the SHG activity is achieved due to the symmetry breaking by chiral Cd(ii)–amine components; however, we found that both optical functionalities are tuned by Cd(ii) complexes. The NLO effect is tuned due to the modulated dipole moments of polar Cd(ii) complexes and their variable arrangement, while a much smaller yet non-negligible contribution was found for Au(i) complexes that are only slightly distorted from the non-polar linear shape. The best-performance SHG is observed for more expanded cpda ligands providing a larger dipole moment of the Cd(ii)–amine units. The weaker SHG was detected for the smaller pda ligand giving a smaller dipole moment of the metal complex. Surprisingly, the material containing enantiopure pda ligands provides significantly weaker SHG than the acentric Cd–Au framework containing the racemate of the ligand. The PL property is tuned by Cd(ii) complexes as they not only induce variable Au–Au distances directly affecting the emission energy but also govern the emission quantum yield (QY). The larger cpda ligand more efficiently quenches the PL due to the increased number of C–H and N–H vibrational modes while the smaller pda ligands lead to much higher QYs above 70%. Thus, the influence of Cd(ii)–amine units on PL is opposite to those observed in SHG. In the context of PL, similarly to the trend found in SHG, the acentric material bearing the racemate of a pda ligand was found to be more efficient than the material with enantiopure pda ligands.

The presented results prove that chiral Cd(ii)–amine complexes play the role of molecular agents stimulating the appearance and optimization of both SHG and PL properties of solids based on aurophilic stacks. A smaller enantiopure amine promotes better PL while a larger amine provides a better SHG. Surprisingly, the best equilibrium between these two optical features is demonstrated for a material containing the racemate of a smaller amine ligand. This was possible due to the crystallization of the racemic mixture of Cd(ii) complexes with dicyanidoaurate(i) ions in the non-centrosymmetric space group. In such a case, the relatively small size of the related ligand contributes to the high efficiency of PL while the more favorable alignment of the related Cd(ii) complexes (due to the different space group) leads to higher SHG efficiency than the observed for the material with enantiopure form of the same ligand. This extraordinary result indicates a promising pathway for further research as racemates of various chiral amines are much easier accessible than their enantiopure analogs. The screening of such a deep pool of racemic amines is thus worth testing when aiming at the optimized NLO-PL response of the materials based on metallophilic aggregates.

The other future pathway may be related to the exploration of chiroptical properties that were preliminarily presented by the circular dichroism effect appearing from the UV-to-vis range thanks to the chiral Cd(ii) complexes and the efficient chirality transfer to the metallophilic stacks based on Au(i) complexes which are responsible for the main light absorption in the indicated range. The work along this line, *e.g.*, in the context of circularly polarized luminescence, is in progress. However, it is important to note that realizing this pathway will rather demand the usage of enantiopure ligands and the subsequent enantiopure Cd(ii) complexes as the compounds containing the racemate, even crystallizing in the non-centrosymmetric space group, are expected to produce rather weak CPL signal (as suggested by negligibly small CD activity for compound of such a type).

## Experimental section

### Materials

All used reagents, including cadmium(ii) chloride hydrate, potassium dicyanoaurate(i), sodium hydroxide, 25% ammonia solution, (*S*)-(−)-propane-1,2-diamine (*S*-pda) dihydrochloride, (*R*)-(+)-propane-1,2-diamine (*R*-pda) dihydrochloride, propane-1,2-diamine (racemic mixture; *rac*-pda), (1*S*,2*S*)-*trans*-cyclopentane-1,2-diamine (*S*-cpda) dihydrochloride, (1*R*,2*R*)-*trans*-cyclopentane-1,2-diamine (*R*-cpda) dihydrochloride, and methanol were obtained from commercial sources and were used without prior purification.

### Synthetic procedures

#### Synthesis of 1-*S* and 1-*R*

The 21 μL (0.26 mmol) portion of 25% ammonia solution was added to the methanolic solution (1 mL) containing the 19.7 mg (0.13 mmol) portion of (*S*)-(−)-propane-1,2-diamine (*S*-pda) dihydrochloride or (*R*)-(+)-propane-1,2-diamine (*R*-pda) dihydrochloride. The resulting solution was added to 12.3 (0.07 mmol) of Cd^II^Cl_2_·*x*H_2_O dissolved in 1 mL of distilled water. The resulting mixture was stirred for several seconds, filtrated, and added to 1 mL of water–methanol (1 : 1, v : v) solution containing 19.3 mg (0.07 mmol) of K[Au^I^(CN)_2_]. The mixture prepared in this way was transferred to a glass vial and allowed to gradually evaporate. White plate-shaped crystals that appeared after about 3 days were collected by filtration, washed with a small portion of water–methanol (1 : 1, v : v) mixture, and dried in the air. Their air-stable composition, {Cd^II^(*S*-pda)_2_[Au^I^(CN)_2_]}[Au^I^(CN)_2_] (**1**-*S*) and {Cd^II^(*R*-pda)_2_[Au^I^(CN)_2_]}[Au^I^(CN)_2_] (**1**-*R*), was determined by single-crystal X-ray diffraction (SC-XRD) studies and confirmed by the CHN elemental analysis and TGA measurements (Fig. S3, ESI†), while the sample purity and phase homogeneity were determined using powder X-ray diffraction (P-XRD) studies (Fig. S12, ESI†). Yield: *ca.* 10 mg, 38% (based on Au). Elemental analysis. Calcd for C_10_H_20_Au_2_Cd_1_N_8_: C, 15.83%; H, 2.66%; N, 14.77%. Found: C, 15.74%; H, 2.68%; N, 14.81% for **1**-*S* and C, 15.86%; H, 2.73%; N, 14.91% for **1**-*R*. IR spectrum (Fig. S2, ESI†): bands situated at 2150 and 2140 cm^−1^ are related to the stretching vibrations of CN^−^ ligands.^77^

#### Synthesis of 2

The 1 mL of water–methanol (1 : 1, v : v) solution containing the 19.3 mg (0.07 mmol) portion of K[Au^I^(CN)_2_] was added to the 2 mL of water–methanol (1 : 1, v : v) solution containing the 11 μL (0.1336 mmol) portion of propane-1,2-diamine (racemic mixture, *rac*-pda) and 12.3 (0.07 mmol) of Cd^II^Cl_2_·*x*H_2_O. This solution was transferred to a glass vial and allowed to gradually evaporate. White block-shaped crystals that appeared after about 3 days were collected by filtration, washed with a water–methanol (1 : 1, v : v) mixture, and dried in the air. Their air-stable composition, {Cd^II^(*S*-pda)_2_[Au^I^(CN)_2_]_2_} (2), was determined by single-crystal X-ray diffraction (SC-XRD) studies and confirmed by the CHN elemental analysis and TGA measurements (Fig. S3, ESI†), while the sample purity and phase homogeneity were determined using powder X-ray diffraction (P-XRD) studies (Fig. S12, ESI†). Yield: 13 mg, 49% (based on Au). Elemental analysis. Calcd for C_10_H_20_Au_2_Cd_1_N_8_: C, 15.83%; H, 2.66%; N, 14.77%. Found: C, 15.62%; H, 2.64%; N, 14.67%. IR spectrum (Fig. S2, ESI†): bands situated at 2160 and 2145 cm^−1^ can be assigned to the stretching vibrations of CN^−^ ligands.^77^

#### Synthesis of 3-*S* and 3-*R*

The syntheses of 3-*S* and 3-*R* were carried out analogously to 1-*S* and 1-*R*, using 10.6 mg (0.26 mmol) of sodium hydroxide instead of ammonia solution, as well as (1*S*,2*S*)-*trans*-cyclopentane-1,2-diamine (*S*-cpda) dihydrochloride or (1*R*,2*R*)-*trans*-cyclopentane-1,2-diamine (*R*-cpda) dihydrochloride instead of (*S*)-(−)-propane-1,2-diamine (*S*-pda) dihydrochloride or (*R*)-(+)-propane-1,2-diamine (*R*-pda) dihydrochloride, respectively. Evaporation of the reaction solution led to the appearance of white plate-shaped crystals, which were filtrated, washed with a water–methanol mixture, and dried in the air. Their air-stable composition, {Cd^II^(*S*-cpda)_2_[Au^I^(CN)_2_]}[Au^I^(CN)_2_] (3-*S*) and {Cd^II^(*R*-cpda)_2_[Au^I^(CN)_2_]}[Au^I^(CN)_2_] (3-*R*), was determined by single-crystal X-ray diffraction (SC-XRD) studies, and confirmed by the CHN elemental analysis and TGA measurements (Fig. S3, ESI†), while the sample purity and phase homogeneity were determined using powder X-ray diffraction (P-XRD) studies (Fig. S12, ESI†). Yield: 14 mg, 49% (based on Au). Elemental analysis. Calcd for C_14_H_24_Au_2_Cd_1_N_8_: C, 20.74%; H, 2.98%; N, 13.82%. Found: C, 20.58%; H, 3.08%; N, 13.73% for 3-*S* and C, 20.11%; H, 2.89%; N, 13.28% for 3-*R*. IR spectrum (Fig. S2, ESI†): bands situated at 2200, 2183, 2156, 2150, and 2134 cm^−1^ correspond to the stretching vibrations of cyanido ligands.^77^

Note that any base can be used to neutralize the ligand hydrochloride but we found the most efficient using ammonia in 1-*S*/1-*R* and sodium hydroxide in 3-*S*/3-*R*. Such conditions allow only little precipitation of cadmium hydroxide and also prevent the formation of other possible coordination compounds, *e.g.*, with oxido/hydroxido ligands.

It is also worth noting the decisive influence of the presence of strong electrolytes on the formation of the presented compounds (Fig. S1, ESI†). Compounds 1-*S* or 1-*R*, as well as 3-*S* or 3-*R*, can be prepared using *S*-pda or *R*-pda and *S*-cpda or *R*-cpda dihydrochlorides, and crystallize always in a pure form. When using a mixture of *S*-pda or *R*-pda ligands, the presence of an additional strong electrolyte (ionic strength of the solution) has a decisive influence on the formation of the crystalline product. In the presence of a strong electrolyte (either formed by the neutralization of the ligand hydrochlorides or added independently), the equimolar mixture of 1-*S* and 1-*R* is formed. However, the situation is different if the neutral rac-pda ligand is used, which causes the formation of 2, containing both forms of optically pure ligands (blue route in Fig. S1a, ESI†). This system can also be obtained by mixing the *S*-pda or *R*-pda dihydrochlorides and removing the strong electrolyte from the solution (orange route at the bottom of Fig. S1a, ESI†). The influence of a strong electrolyte on the inhibition of crystallization of a compound containing both forms of optically pure ligands takes place only in the case of propane-1,2-diamine ligand (Fig. S1a, ESI†), while in the case of cyclopentane-1,2-diamine, pure 3-*S* or 3-*R* systems or their equimolar mixture are always formed (Fig. S1b, ESI†). No other crystalline forms, such as analogous to 2, are formed.

### X-ray crystallography

The single-crystal X-ray diffraction data for 1-*S*, 1-*R*, 2, 3-*S*, and 3-*R* were collected both at 100(2) and 300(2) K using a Bruker D8 Quest diffractometer equipped with a Photon50 CMOS detector, Mo Kα (0.71073 Å) irradiation source, a graphite monochromator, equipped also with an Oxford Cryostream cooling system. The selected single crystals were taken directly from the mother solution, dispersed with Apiezon N grease, and measured at 100(2) K. The appropriate crystal was mounted on MicroMounts^TM^ and measured firstly at 100(2) K. After finishing the measurement, the crystal was slowly heated to 300(2) K. After the stabilization of the temperature for *ca.* 30 min, the measurement at 300(2) K was performed. The absorption correction was executed using the TWINABS program.^109^ All presented crystal structures were solved by an intrinsic phasing method using SHELXT-2014/5 and refined by a full-matrix least-squares technique on *F*^2^ using the SHELXL–2018/3 program within the WinGX system.^110,111^ Non-hydrogen atoms were refined anisotropically while hydrogen atoms for *S*-pda, *R*-pda, *rac*-pda, *S*-cpda, and *R*-cpda were calculated in their idealized positions and further refined using a riding model. High-quality data obtained within the SC-XRD experiments requires applying only a few restraints for thermal ellipsoids (of ISOR type) for C atoms of diamine ligands in 2 and 3-*S* to ensure the convergence of the refinement process, achieve satisfactory refinement parameters, and keep the proper geometry of the ligands.

The reference CCDC numbers for 1-*S*, 1-*R*, 2, 3-*S*, and 3-*R* for the crystal structures at 100(2) K are 2309223, 2309225, 2309227, 2309229, and 2309331, respectively, while for the crystal structures at 300(2) K are 2309224, 2309226, 2309228, 2309230, and 2309232, respectively. Details of crystal data and structure refinement are summarized in Tables S1–S3 (ESI†) while the representative structural parameters are gathered in Tables S4–S6 (ESI†). Structural figures were prepared using the Mercury 2022.1.0 software.

The powder X-ray diffraction (P-XRD) data were collected using a Bruker D8 Advance Eco powder diffractometer equipped with a Cu Kα (1.5419 Å) radiation source. The P-XRD measurements were conducted at room temperature for the polycrystalline samples inserted into a glass capillary using the appropriate experimental setup for rotating capillary (preventing problems with the preferred orientation of crystallites).

### Physical techniques

The CHN elemental analyses were measured using an Elemental Vario Micro Cube analyzer. Infrared (IR) absorption spectra were measured on selected crystals using a Thermo Scientific Nicolet iN10 FTIR spectrometer in the range 4000–700 cm^−1^. The UV-vis absorption spectra were collected in the range of 260–700 nm using a Shimadzu UV-3600 double-beam spectrophotometer. Circular dichroism (CD) spectra were collected using the JASCO J-810 spectropolarimeter. They were measured on the powder samples mixed with a small amount of paraffin oil and placed between two quartz plates. The TGA curves were collected under a nitrogen atmosphere using a NETZSCH TG209 F1 Libra thermogravimetric analyzer with Al pans as holders in the 20–400 °C temperature range with a heating rate of 1 °C min^−1^. Second harmonic generation (SHG) experiments were performed using a homemade optical setup, described by us in detail previously.^50^ Briefly, a 1040 nm femtosecond laser was used as incident fundamental light, which was used to irradiate the surface of the sample, prepared in the powder pellet form, and placed in a glass holder. The resulting SHG signal was detected in a reflection mode by a photomultiplier tube. To verify the SHG activity, each sample was characterized by both the power dependence and the wavelength-dependence measurements. For the first measurement, the incident laser intensity was tuned with a linear increment from zero, while for the latter measurement, the detection wavelength was adjusted by the monochromator. To quantify the SHG intensities generated by the investigated samples, a potassium dihydrogen phosphate (KDP) powder pellet was used as a reference sample. The solid-state photoluminescent spectra, including emission and excitation spectra, were measured using an FS5 spectrofluorometer (Edinburgh Instruments) equipped with a Xe (150 W) arc lamp as an excitation source and a Hamamatsu photomultiplier of the R928P type applied as a detector. Emission lifetime measurements were conducted on the FS5 spectrofluorometer using a time-correlated single photon counting (TCSPC) method with picosecond pulsed light emitting diodes, EPLED-340 (336.2 nm) for 3-*S* and 3-*R* and EPLED-380 (374.2 nm) for 1-*S*, 1-*R*, and 2. For photoluminescent measurements at 300(2) and 77(2) K, the powder sample of the selected material was inserted in the quartz tube placed in the optical dewar filled with liquid nitrogen. Absolute luminescence quantum yields were determined by a direct excitation method using an integrating sphere module for FS5, the details are described in Ref. 82. Luminescent background corrections were performed within the Fluoracle software (Edinburgh Instruments).

## Data availability

The data supporting this article have been included as part of the ESI.† Crystallographic data for 1-*S*, 1-*R*, 2, 3-*S*, and 3-*R* has been deposited at the CCDC database under 2309223, 2309225, 2309227, 2309229, and 2309331 numbers for the crystal structures at 100(2) K, respectively, while for the crystal structures at 300(2) K under 2309224, 2309226, 2309228, 2309230, and 2309232 numbers. The other data can be obtained from the corresponding author.

## Conflicts of interest

There are no conflicts to declare.

## Supplementary Material

TC-012-D4TC01042F-s001

TC-012-D4TC01042F-s002
