# Supplementary material for: Chiral cadmium–amine complexes for stimulating non-linear optical activity and photoluminescence in solids based on aurophilic stacks
Source: J Mater Chem C Mater. 2024 Aug 23;12(37):14964–77. doi: 10.1039/d4tc01042f (PMC11343038; doi:10.1039/d4tc01042f)
Supplement: TC-012-D4TC01042F-s001 [file TC-012-D4TC01042F-s001.pdf]

Supplementary Information (SI) for Journal of Materials Chemistry C.
This journal is © The Royal Society of Chemistry 2024

SUPPORTING INFORMATION

Chiral cadmium-amine complexes for stimulating non-linear optical activity and photoluminescence in solids based on aurophilic stacks

Kseniia Boidachenko,[a,b] Michal Liberka,[a,b] Junhao Wang,[c] Hiroko Tokoro,[c] Shin-ichi Ohkoshi,[d] and Szymon Chorazy*,[a]

[a]Faculty of Chemistry, Jagiellonian University, Gronostajowa 2, 30-387 Krakow, Poland; [b]Doctoral School of Exact and Natural Sciences, Jagiellonian University, Łojasiewicza 11, 30-348 Kraków, Poland; [c]Department of Materials Science, Institute of Pure and Applied Sciences, University of Tsukuba, 1-1-1 Tennodai, Tsukuba, Ibaraki 305-8573, Japan; [d]Department of Chemistry, School of Science, The University of Tokyo, 7-3-1 Hongo, Bunkyo-ku, Tokyo 113-0033, Japan. *Corresponding author: simon.chorazy@uj.edu.pl

Summary of various routes towards the syntheses of **1**-*S*, **1**-*R*, **2**, **3**-*S*, and **3**-*R*. (Fig. S1)	**S2**
Infrared (IR) absorption spectra of **1**-*S*, **1**-*R*, **2**, **3**-*S*, and **3**-*R*, with their interpretation. (Fig. S2)	**S3**
Thermogravimetric (TG) curves of **1**-*S*, **1**-*R*, **2**, **3**-*S*, and **3**-*R*, with their interpretation. (Fig. S3)	**S4**
Summaries of crystal data and structure refinement for **1**-*S*, **1**-*R*, **2**, **3**-*S*, and **3**-*R*. (Table S1−S3)	**S5−S7**
The representative views of the coordination framework of **1**-*S* and **1**-*R* at 100(2) and 300(2) K, shown along three main crystallographic axes. (Fig. S4 and S5)	**S8−S9**
Detailed structural views of the crystal structures of **1**-*S* and **1**-*R* at 100(2) K and 300(2) K. (Fig. S6)	**S10**
Detailed structure parameters of **1**-*S* and **1**-*R* at 100(2) K and 300(2) K. (Table S4)	**S11**
The representative views of the coordination framework of **2** at 100(2) K and 300(2) K. (Fig. S7)	**S12**
Detailed structural views of the crystal structure **2** at 100(2) K and 300(2) K. (Fig. S8)	**S13**
Detailed structure parameters of **2** at 100(2) K and 300(2) K. (Table S5)	**S14**
The representative views of the coordination framework of **3**-*S* and **3**-*R* at 100(2) and 300(2) K, shown along three main crystallographic axes. (Fig. S9 and S10)	**S15−S16**
Detailed structural views of the crystal structures of **3**-*S* and **3**-*R* at 100(2) K and 300(2) K. (Fig. S11)	**S17**
Detailed structure parameters of **3**-*S* and **3**-*R* at 100(2) K and 300(2) K. (Table S6)	**S18–S19**
Results of Continuous Shape Measure (CShM) analyses for Cd(II) and Au(I) in **1**-*S*, **1**-*R*, **2**, **3**-*S*, and **3**-*R*, together with the related comment. (Tables S7 and S8)	**S20–S21**
Powder X-ray diffraction (P-XRD) patterns of **1**-*S*, **1**-*R*, **2**, **3**-*S*, and **3**-*R*. (Fig. S12)	**S22**
The wavelength-dependences of the SHG signal and the dependence of the SHG light intensity on the excitation light intensity for KDP (reference), **1**-*S*, **1**-*R*, **2**, **3**-*S*, and **3**-*R*. (Fig. S13 and S14)	**S23–S24**
Comparison of the SHG intensities generated by compounds **1**-*S*, **1**-*R*, **2**, **3**-*S*, and **3**-*R* with the KDP reference. (Table S9)	**S25**
Results of dipole moment calculations of Cd(II) complexes in **1**-*S*, **1**-*R*, **2**, **3**-*S*, and **3**-*R*. (Table S10)	**S26**
Results of dipole moment calculations of Au(I) complexes in **1**-*S*, **1**-*R*, **2**, **3**-*S*, and **3**-*R*. (Table S11)	**S27–S28**
The directions of dipole moment vectors in the {CdN_6} distorted octahedrons in **1**-*S*, **2**, and **3**-*S*, and the schematic presentation of the directions of dipole moment vectors of all metal complexes as well as favorable and unfavorable dipole-dipole interactions **1**-*S* and **2**. (Fig. S15)	**S29**
Comparison of estimated dipole moments with the SH intensities and the CShM analyses in the set of obtained materials. (Fig. S16)	**S30**
Comment on dipole moment calculations.	**S31**
Solid-state UV-vis and circular dichroism absorption spectra for **1**-*S*, **1**-*R*, **2**, **3**-*S*, and **3**-*R*. (Fig. S17)	**S32**
Selected curves of solid-state photoluminescent properties of **1**-*S* and **1**-*R*. (Fig. S18)	**S33**
Selected curves of solid-state photoluminescent properties of **2**. (Fig. S19)	**S34**
Selected curves of solid-state photoluminescent properties of **3**-*S* and **3**-*R*. (Fig. S20)	**S35**
Selected parameters of the luminescent properties of **1**-*S*, **1**-*R*, **2**, **3**-*S*, and **3**-*R*. (Tables S12 and S13)	**S36−S37**
Literature to the Supporting Information.	**S38**

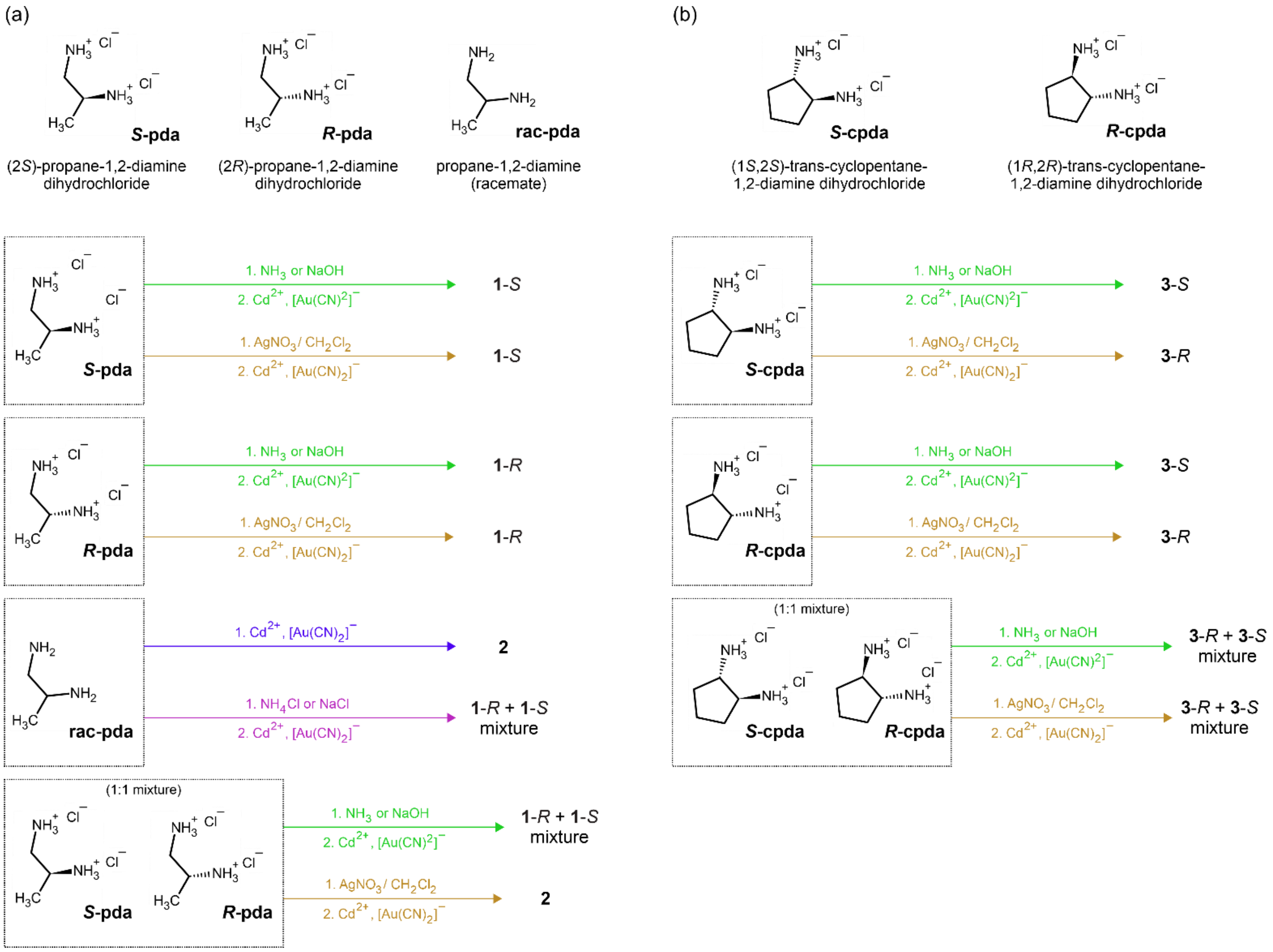

Fig. S1. Schematic presentation of various routes towards the syntheses of **1**-*S*, **1**-*R*, and **2** (*a*), as well as **3**-*S*, and **3**-*R* (*b*) materials.

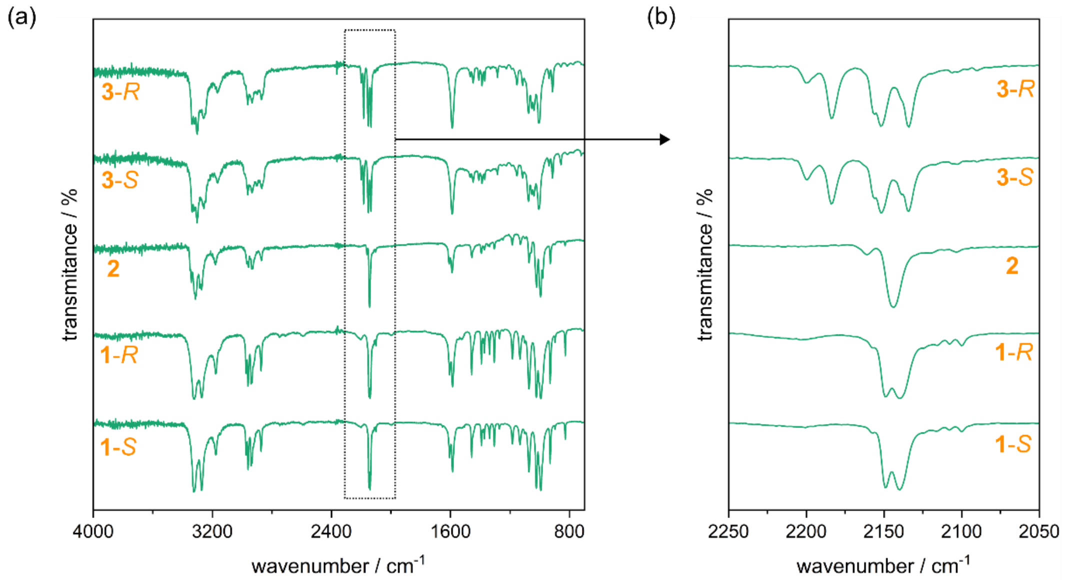

Fig. S2. Infrared (IR) absorption spectra of **1**-*S*, **1**-*R*, **2**, **3**-*S*, and **3**-*R* presented in the broad 4000–700 cm^{-1} region (*a*) and the 2250–2050 cm^{-1} region assignable to cyanido stretching vibrations (*b*).

Comment to Fig. S2: The IR spectra of **1**-*S*, **1**-*R*, **2**, **3**-*S*, and **3**-*R* are very similar. In all samples, the wide absorption above 2850 cm^{-1}, as well as the extensive area of absorption bands in the 1500–700 cm^{-1} range is related to the stretching and skeletal vibrations of amine ligands. Moreover, the N–H bending vibration of primary amines is observed in the region of 1615−1565 cm^{-1}. In the range of 2220−2120 cm^{-1}, characteristic peaks related to stretching vibrations of cyanido ligands can be observed. The higher energy bands above 2145 cm^{-1} can be attributed to vibrations of Cd^{II}–N≡C–Au^{I} cyanido bridges, while the lower energy peaks to terminal cyanido ligands. More absorption bands in this range for **3**-*S* and **3**-*R* are associated with a greater number of various molecular bridging modes as their crystal structures include both bent (weaker) and almost linear (stronger) cyanido bridges (Fig. 2), appearing in the lower and higher energy ranges, respectively. Due to the presence of almost linear Cd^{II}–N≡C–Au^{I} modes in **3**-*S* and **3**-*R*, the related stretching vibrations are shifted towards higher energies when compared to **1**-*S*, **1**-*R*, and **2**, in which only one kind of bent molecular cyanido bridges can be observed. The gathered spectroscopic results are in line with structural models of the respective materials (Fig. 1 and 2) and previous works on related dicyanidoaurate(I)-based compounds.[S1–S3]

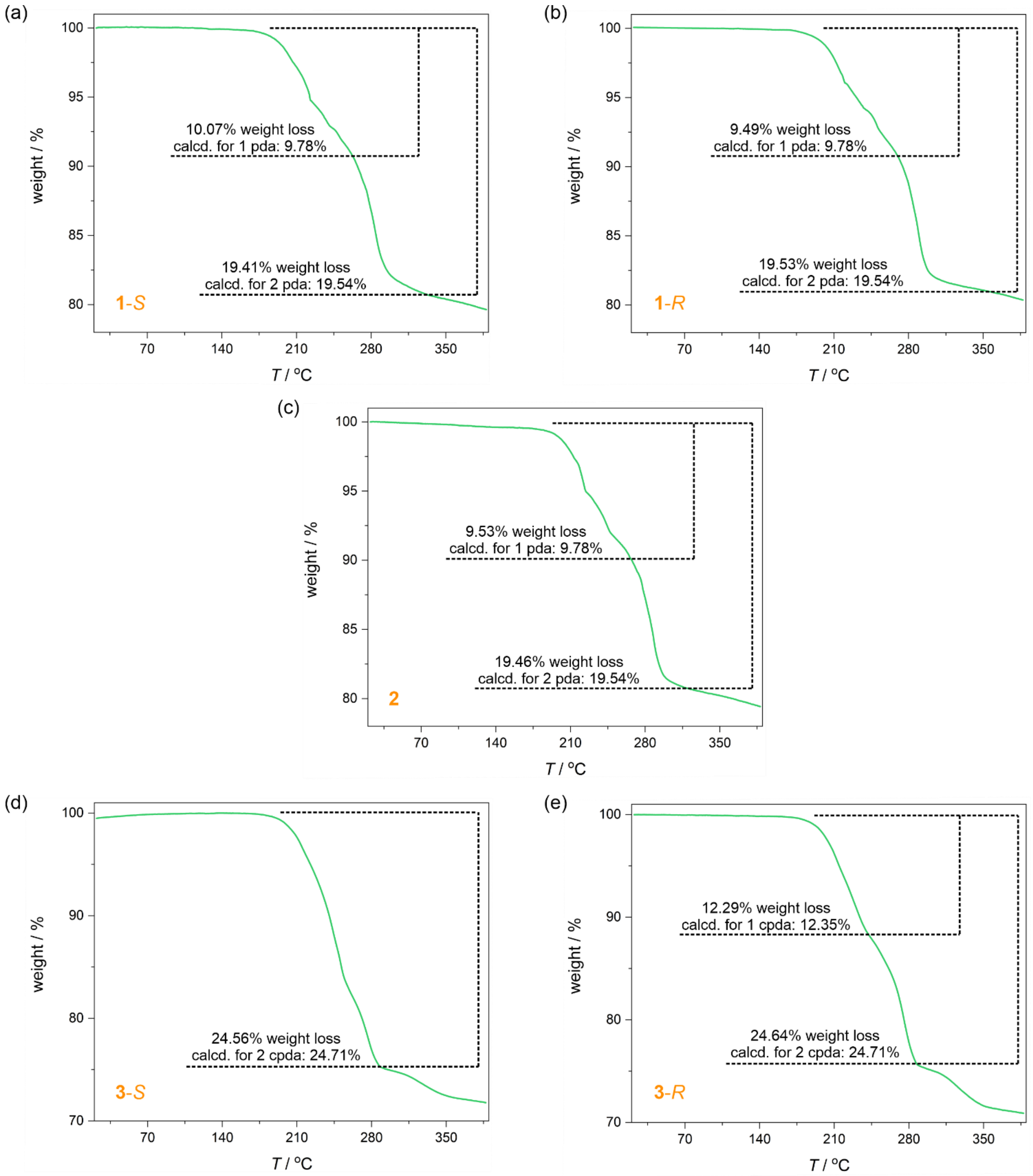

Fig. S3. Thermogravimetric (TG) curves collected in the temperature range of 20–390 °C for **1**-*S* (*a*), **1**-*R* (*b*), **2** (*c*), **3**-*S* (*d*), and **3**-*R* (*e*). The steps related to the loss of diamine ligands are depicted.

Comment to Fig. S3: All presented compounds are stable with heating up to ca. 200 °C when the gradual decrease of the sample mass is detected. The two-step weight loss in **1**-*S*, **1**-*R*, and **2** can be assigned to the removal of two pda ligands per the {$Cd^{II}Au^{I}_2$} unit (theoretical values are 9.8% for one and 19.5% for two pda molecules). A similar two-step decrease in **3**-*R* can be attributed to the removal of the two cpda ligands, while in **3**-*S* there is a single abrupt decrease corresponding to the removal of two cpda molecules per the {$Cd^{II}Au^{I}_2$} unit (theoretical values are 12.4% for one and 24.7% for two cpda molecules).

Table S1. Crystal data and structure refinement parameters for **1**-*S* and **1**-*R* for the SC-XRD measurements performed at 100(2) and 300(2) K.

compound	**1**-*S*		**1**-*R*	
formula	$C_{10}H_{20}Au_2Cd_1N_8$		$C_{10}H_{20}Au_2Cd_1N_8$	
formula weight / g·mol^{-1}	758.67		758.67	
λ / Å	0.71073 (Mo Kα)		0.71073 (Mo Kα)	
T / K	100(2)	300(2)	100(2)	300(2)
crystal system	monoclinic	monoclinic	monoclinic	monoclinic
space group	$C2$ (no. 5)	C2 (no. 5)	$C2$ (no. 5)	$C2$ (no. 5)
a / Å	15.5698(8)	15.7042(9)	15.5671(14)	15.711(4)
b / Å	6.3006(3)	6.4059(4)	6.3024(5)	6.4114(14)
c / Å	9.9989(5)	9.9622(5)	9.9993(9)	9.978(2)
α / deg	90	90	90	90
β / deg	113.660(2)	113.882(2)	113.676(3)	113.935(9)
γ / deg	90	90	90	90
V / Å^3	898.43(8)	916.39(9)	898.46(14)	918.7(4)
Z	2	2	2	2
calcd. density / g·cm^{-1}	2.804	2.750	2.804	2.743
abs. coeff. / cm^{-1}	17.467	17.125	17.466	17.082
F(000)	684	684	684	684
Θ range / deg	2.830−24.989	2.813−25.025	2.830−24.986	2.233−25.019
limiting indices	$-18 < h < 18$<br>$-7 < k < 7$<br>$-11 < l < 11$	$-18 < h < 18$<br>$-7< k < 7$<br>$-11 < l < 11$	$-18 < h < 18$<br>$-7< k < 7$<br>$-11 < l < 11$	$-18 < h < 18$<br>$-7< k < 7$<br>$-11 < l < 11$
collected refl.	6299	5217	5026	7774
R_{int}	0.0251	0.0326	0.0264	0.0476
completeness / %	99.9	99.8	99.9	99.9
data/restraints/parameters	1564/1/97	1600/1/97	1571/1/97	1621/1/97
Flack parameter	0.064(11)	0.112(15)	0.097(12)	0.065(17)
GOF on F^2	1.055	1.068	1.094	1.059
final R indices<br>R_1 [$I > 2\sigma(I)$]; wR_2 [all data]	R_1 = 0.0151<br>wR_2 = 0.0349	R_1 = 0.0272<br>wR_2 = 0.0631	R_1 = 0.0174<br>wR_2 = 0.0417	R_1 = 0.0267<br>wR_2=0.0584
diff. peak and hole / e·Å^{-3}	1.542 and −1.813	2.137 and −3.183	1.991 and −2.533	0.733 and −1.849

Table S2. Crystal data and structure refinement parameters for **2** for the SC-XRD measurements performed at 100(2) and 300(2) K.

compound	**2**	
formula	$C_{10}H_{20}Au_2Cd_1N_8$	
formula weight / g·mol^{-1}	758.67	
λ / Å	0.71073 (Mo Kα)	
T / K	100(2)	300(2)
crystal system	orthorhombic	orthorhombic
space group	*Aba*2 (no. 41)	*Aba*2 (no. 41)
a / Å	18.5216(11)	18.457(5)
b / Å	15.1445(9)	15.272(4)
c / Å	6.3905(4)	6.4830(16)
α / deg	90	90
β / deg	90	90
γ / deg	90	90
V / Å^3	1792.54(19)	1827.4(8)
Z	4	4
calcd. density / g·cm^{-1}	2.811	2.758
abs. coeff. / cm^{-1}	17.509	17.175
F(000)	1368	1368
Θ range / deg	2.906−26.461	2.667−26.386
limiting indices	$-23 < h < 23$<br>$-18 < k < 18$<br>$-8 < l < 7$	$-23 < h < 23$<br>$-18 < k < 18$<br>$-8 < l < 8$
collected refl.	10151	13480
R_{int}	0.0448	0.0441
completeness / %	99.8	99.9
data/restraints/parameters	1814/13/96	1868/1/96
Flack parameter	0.088(8)	0.065(6)
GOF on F^2	1.069	1.042
final R indices<br>R_1 [$I > 2\sigma(I)$]; wR_2 [all data]	R_1 = 0.0214<br>wR_2 = 0.0440	R_1 = 0.0199<br>wR_2 = 0.0406
diff. peak and hole / e·Å^{-3}	0.883 and −0.702	0.433 and −0.623

Table S3. Crystal data and structure refinement parameters for **3**-*S* and **3**-*R* for the SC-XRD measurements performed at 100(2) and 300(2) K.

compound	**3**-*S*		**3**-*R*	
formula	$C_{14}H_{24}Au_2Cd_1N_8$		$C_{14}H_{24}Au_2Cd_1N_8$	
formula weight / g·mol^{-1}	810.74		810.74	
λ / Å	0.71073 (Mo Kα)		0.71073 (Mo Kα)	
T / K	100(2)	300(2)	100(2)	300(2)
crystal system	orthorhombic	orthorhombic	orthorhombic	orthorhombic
space group	$P2_12_12$ (no. 18)	$P2_12_12$ (no. 18)	$P2_12_12$ (no. 18)	$P2_12_12$ (no. 18)
a / Å	15.4474(8)	15.6818(4)	15.4338(11)	15.6792(5)
b / Å	19.7190(10)	19.6256(5)	19.7017(15)	19.6095(6)
c / Å	6.6061(3)	6.6615(2)	6.6040(4)	6.6587(2)
α / deg	90	90	90	90
β / deg	90	90	90	90
γ / deg	90	90	90	90
V / Å^3	2012.27(17)	2050.18(10)	2008.1(2)	2047.29(11)
Z	4	4	4	4
calcd. density / g·cm^{-1}	2.676	2.627	2.682	2.630
abs. coeff. / cm^{-1}	15.607	15.318	15.639	15.34
F(000)	1480	1480	1480	1480
Θ range / deg	2.637−25.026	2.448−25.027	2.453−25.010	2.450−25.026
limiting indices	$-18 < h < 18$<br>$-23 < k < 23$<br>$-7 < l < 7$	$-18 < h < 18$<br>$-23 < k < 23$<br>$-7 < l < 7$	$-18 < h < 18$<br>$-23 < k < 23$<br>$-7 < l < 7$	$-18 < h < 18$<br>$-23 < k < 23$<br>$-7 < l < 7$
collected refl.	23176	23758	32942	23673
R_{int}	0.0330	0.0455	0.0519	0.0306
completeness / %	99.9	99.9	99.9	99.6
data/restraints/parameters	3519/3/227	3608/3/234	3533/9/227	3596/3/232
Flack parameter	0.056(5)	0.053(8)	0.048(5)	0.060(7)
GOF on F^2	1.134	1.083	1.092	1.083
final R indices R_1 [$I > 2\sigma(I)$]; wR_2 [all data]	R_1 = 0.0168<br>wR_2 = 0.0320	R_1 = 0.0265<br>wR_2 = 0.0523	R_1 = 0.0200<br>wR_2 = 0.0405	R_1 = 0.0290<br>wR_2 = 0.0595
diff. peak and hole / e·Å^{-3}	0.599 and −0.596	0.602 and −1.100	1.326 and −0.852	1.516 and −1.120

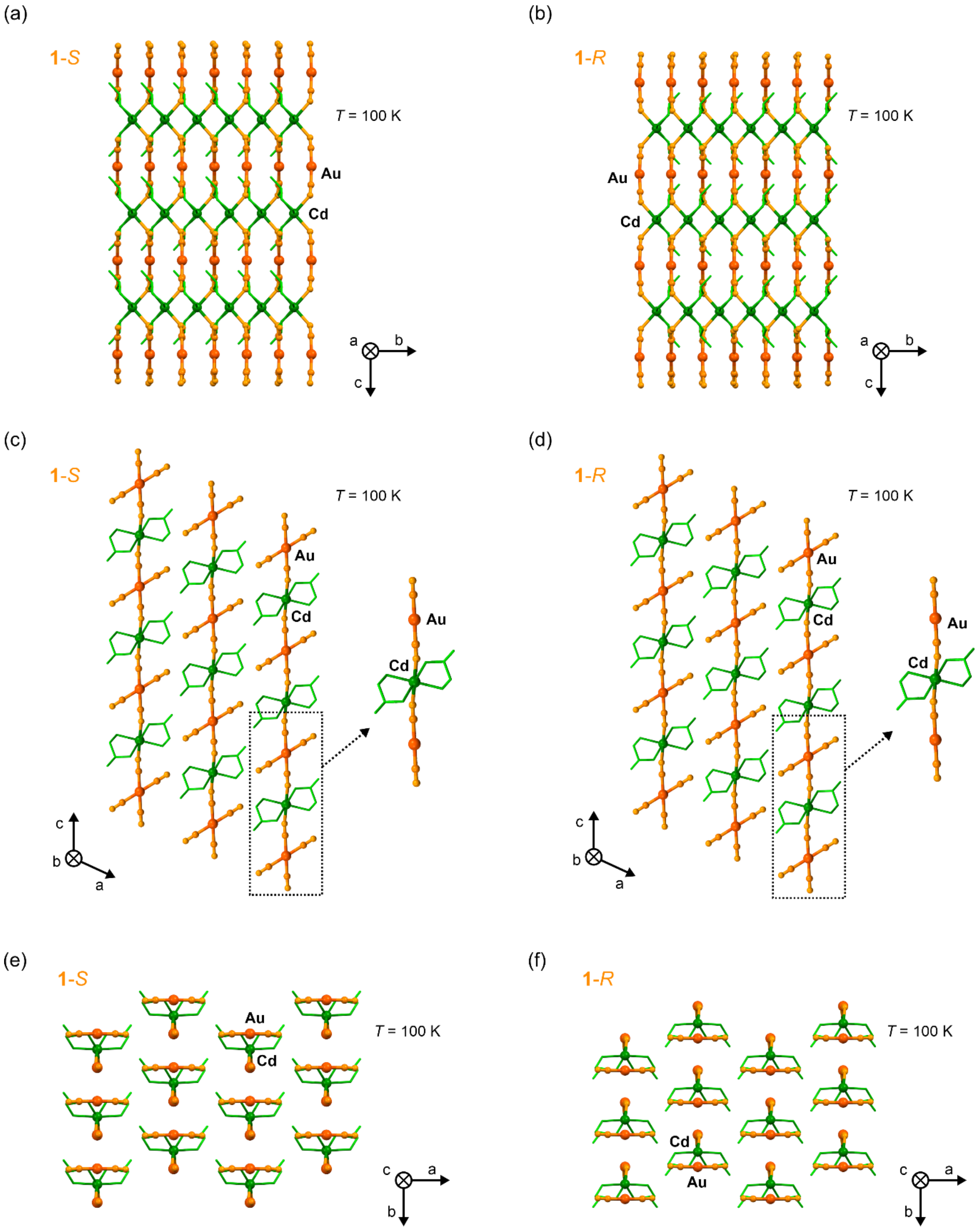

Fig. S4. The representative views of the coordination framework of **1**-*S* (left panel, i.e., *a*, *c*, and *e* parts) and **1**-*R* (right panel, i.e., *b*, *d*, and *f* parts) for the crystal structures determined at 100(2) K, presented along the main **a**, **b**, and **c** crystallographic axes (parts *a*–*b*, *c*–*d*, and *e*–*f*, respectively). Hydrogen atoms were omitted for clarity. Colors: green with various hues = Cd centers with amine ligands attached to them, orange with various hues = Au centers with cyanido ligands.

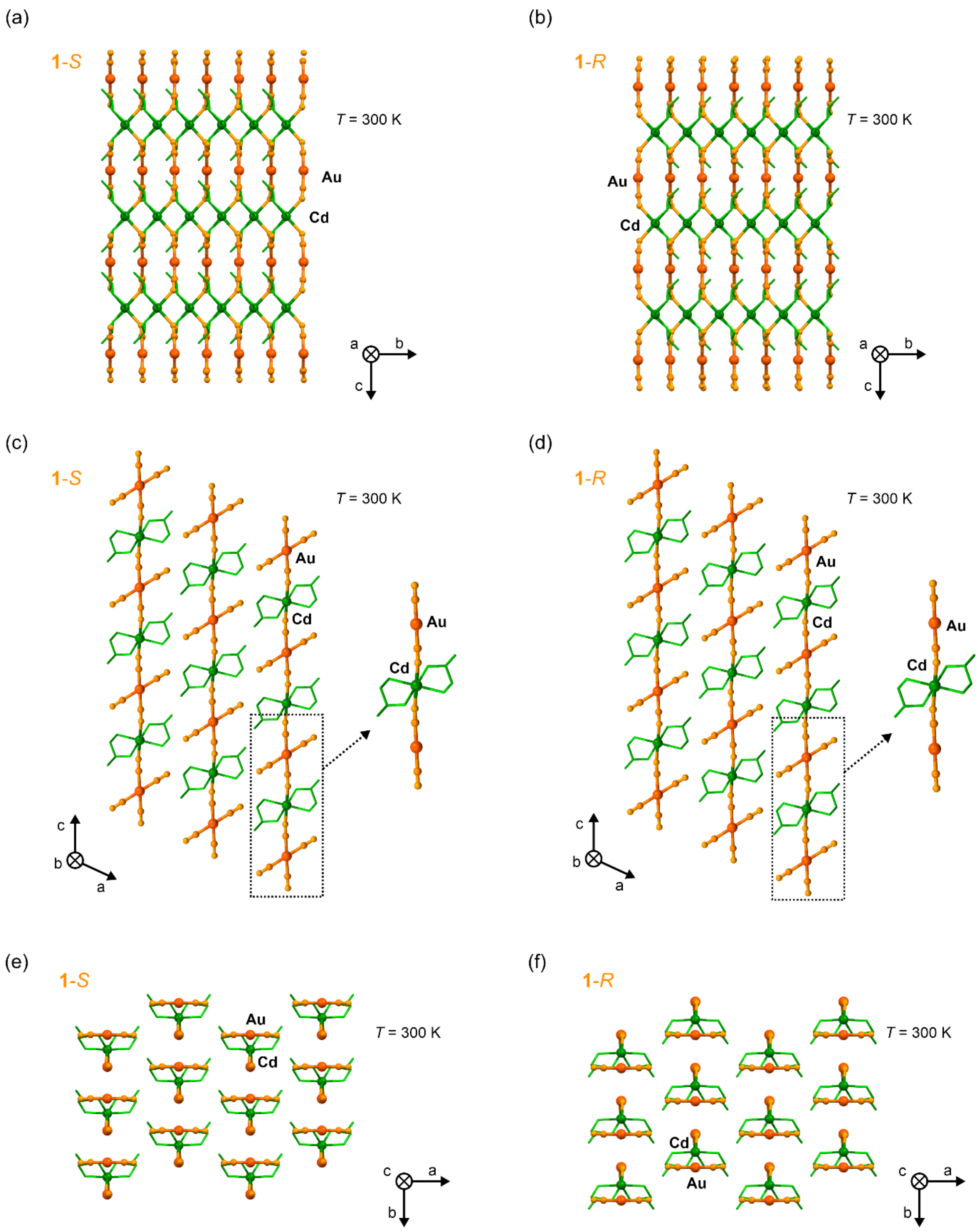

Fig. S5. The representative views of the coordination framework of **1**-*S* (left panel, i.e., *a*, *c*, and *e* parts) and **1**-*R* (right panel, i.e., *b*, *d*, and *f* parts) for the crystal structures determined at 300(2) K, presented along the main **a**, **b**, and **c** crystallographic axes (parts *a*–*b*, *c*–*d*, and *e*–*f*, respectively). Hydrogen atoms were omitted for clarity. Colors: green with various hues = Cd centers with amine ligands attached to them, orange with various hues = Au centers with cyanido ligands.

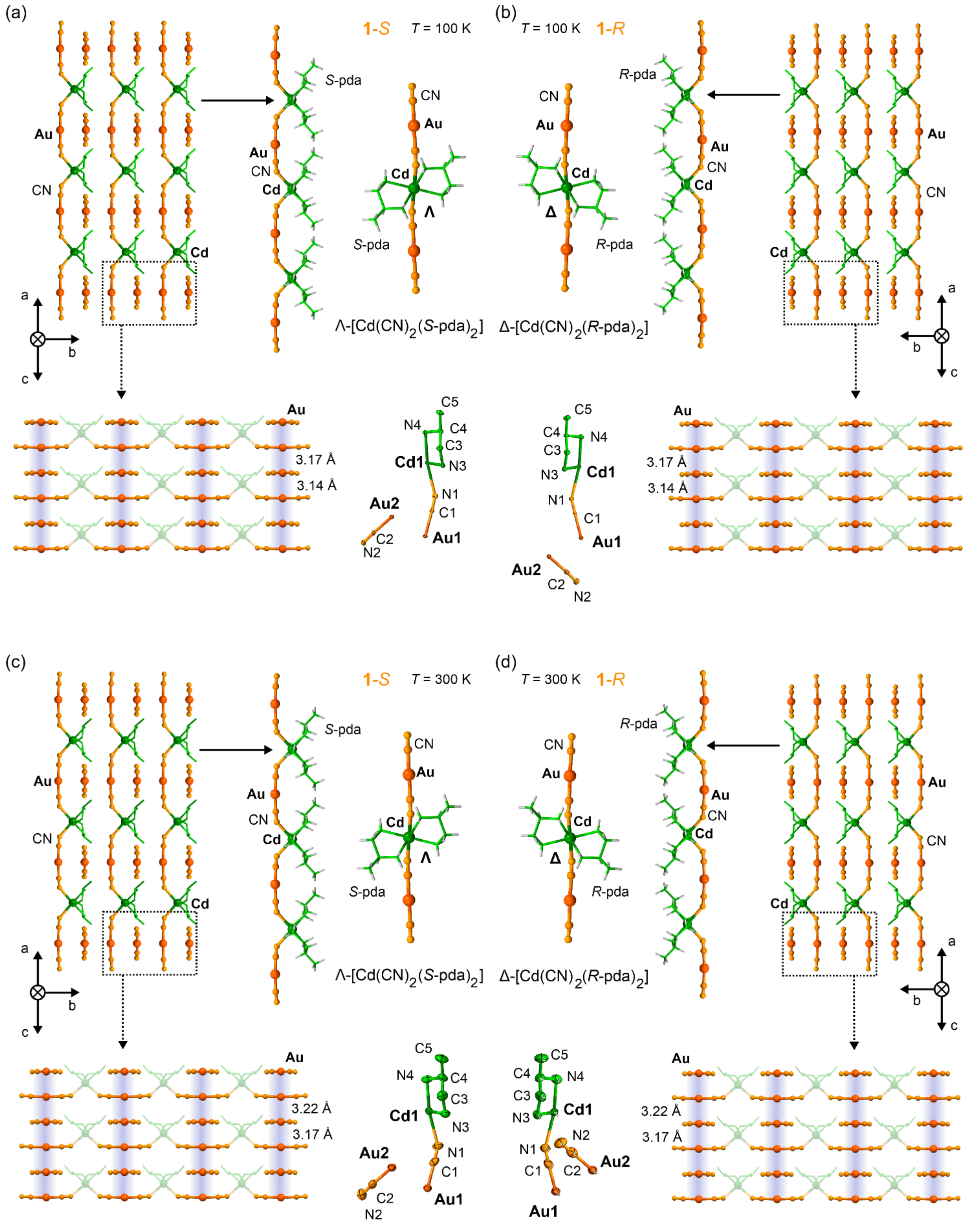

Fig. S6. Detailed structural views of **1**-*S* (left panel, i.e., *a* and *c* parts) and **1**-*R* (right panel, i.e., *b* and *d* parts) at 100(2) K (upper part, i.e., *a* and *b* parts) and 300(2) K (bottom part, i.e., *c* and *d* parts), including the detailed presentation of cyanido-bridged {$Cd^{II}Au^{I}$}-based chains with a demonstration of the Cd(II) coordination sphere (i.e., *cis*-*Λ*/*Δ*-[Cd^{II}(*μ*-NC)$_2$(*S*-/*R*-pda)$_2$]), the visualization of Au···Au metallophilic interaction pattern with depicted intermetallic distances, and the asymmetric unit with the labeling scheme for selected symmetrically independent atoms. Thermal ellipsoids for the asymmetric unit are presented at the 50% probability level. Hydrogen atoms in some views were omitted for clarity. Colors: green with various hues = Cd centers with amine ligands attached to them, orange with various hues = Au centers with cyanido ligands.

Table S4. Comparison of detailed crystal structure parameters of **1**-*S* and **1**-*R* at 100(2) and 300(2) K.

compound	**1**-*S*		**1**-*R*	
T / K	100(2)	300(2)	100(2)	300(2)
selected bond distances and angles in *cis*-[Cd1(*μ*-NC)$_2$(pda)$_2$] complexes / Å, °				
Cd1−N1	2.434(8)	2.442(12)	2.439(7)	2.449(12)
Cd1−N3	2.303(5)	2.293(7)	2.302(4)	2.304(8)
Cd1−N4	2.366(8)	2.367(12)	2.367(7)	2.376(12)
N1−Cd1−N1	99.9(3)	99.0(6)	100.0(3)	99.2(6)
N3−Cd1−N3	173.9(5)	174.8(9)	174.0(5)	174.9(10)
N4−Cd1−N4	100.9(3)	99.7(6)	100.5(3)	99.6(6)
N1−Cd1−N3	85.0(3)/99.0(3)	84.1(5)/99.3(6)	85.0(3)/98.8(3)	84.5(5)/98.8(5)
N1−Cd1−N4	83.0(2)/160.5(3)	84.3(4)/159.5(5)	83.1(2)/160.4(2)	84.2(4)/159.7(4)
N3−Cd1−N4	75.5(3)/100.5(3)	75.4(4)/101.2(5)	75.4(2)/100.7(3)	75.1(4)/101.5(5)
intermetallic distances and other structural parameters / Å, °				
Au1−Cd1	5.282(11)	5.275(18)	5.282(11)	5.280(20)
C1−N1−Cd1	142.9(5)	143.4(8)	143.3(5)	143.3(9)
Au1−Au2	3.135(11)/3.165(11)	3.186(18)/3.220(18)	3.137(11)/3.165(11)	3.187(20)/3.224(20)
hydrogen bond parameters (within a single aurophilic-interaction-based layer) / Å, °				
N1···N4<br>N1···H4A<br>N1···H4A−N4	3.32<br>2.52<br>150.3	3.41<br>2.60<br>150.5	3.33<br>2.52<br>151.4	3.40<br>2.60<br>149.8
N2···N3<br>N2···H3B<br>N2···H3B−N3	3.09<br>2.24<br>159.2	3.11<br>2.27<br>157.2	3.09<br>2.23<br>159.9	3.12<br>2.28<br>156.4
Au2···N4<br>Au2···H4B<br>Au2···H4B−N4	3.55<br>2.69<br>164.0	3.55<br>2.69<br>164.7	3.55<br>2.68<br>164.0	3.56<br>2.69<br>165.2
hydrogen bond parameters (between aurophilic-interaction-based layers) / Å, °				
N2···N3<br>N2···H3<br>N2···H3A−N3	3.02<br>2.23<br>148.3	3.04<br>2.25<br>147.2	3.02<br>2.22<br>148.7	3.03<br>2.24<br>156.4

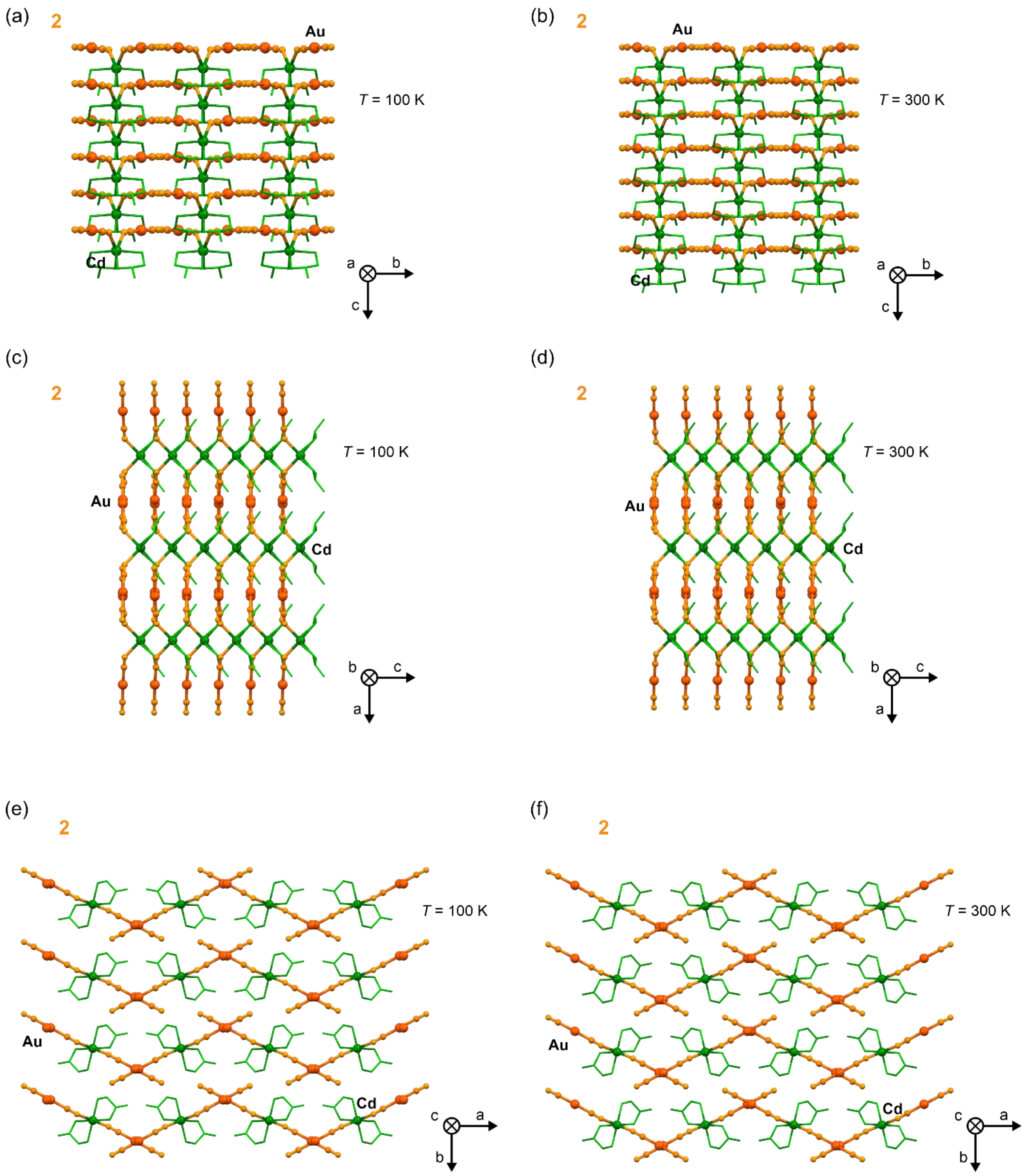

Fig. S7. The representative views of the coordination framework of **2** at 100(2) K (left panel, i.e., *a*, *c*, and *e* parts) and 300(2) K (right panel, i.e., *b*, *d*, and *f* parts), presented along the main **a**, **b**, and **c** crystallographic axes (parts *a*–*b*, *c*–*d*, and *e*–*f*, respectively). Hydrogen atoms were omitted for clarity. Colors: green with various hues = Cd centers with amine ligands attached to them, orange with various hues = Au centers with cyanido ligands.

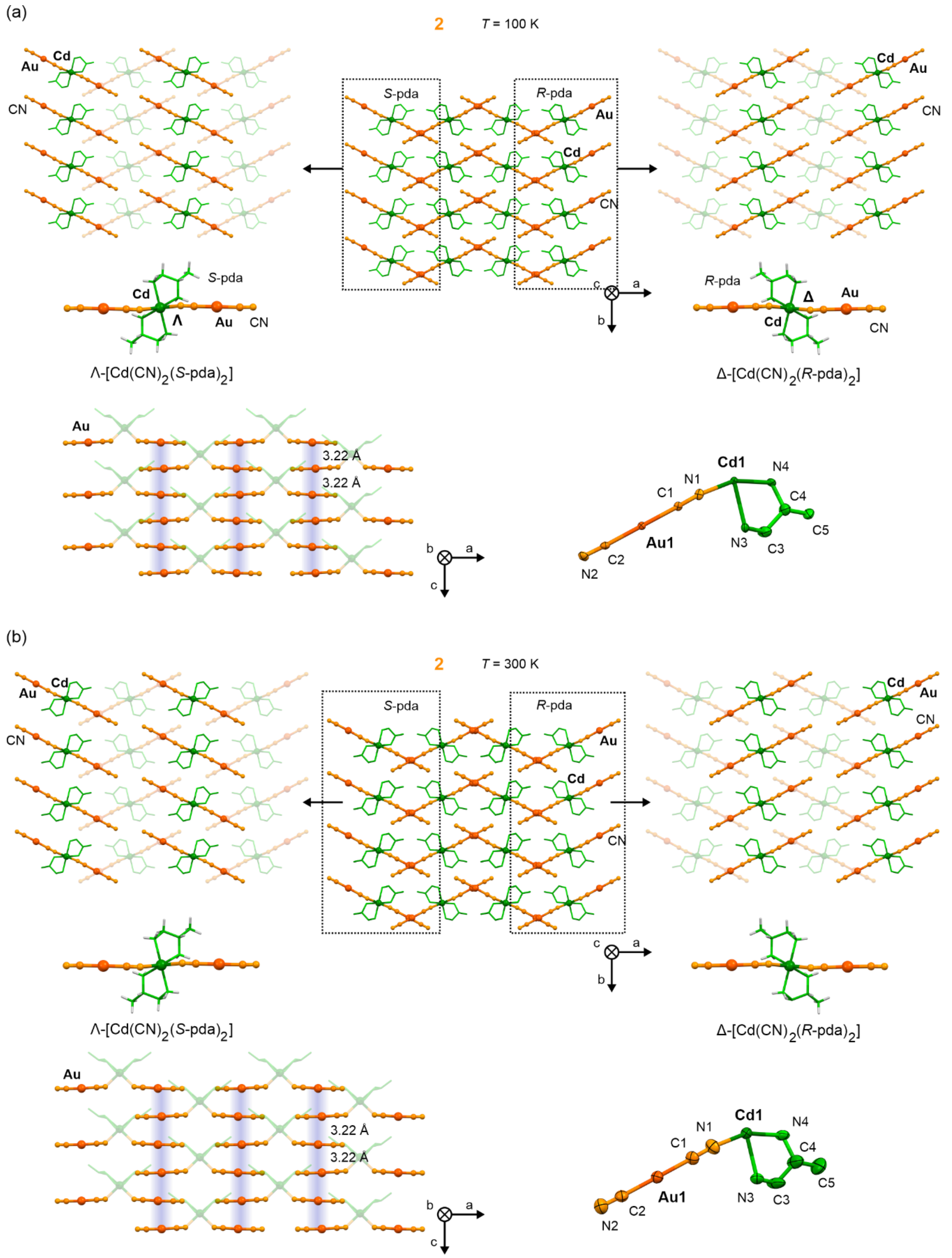

Fig. S8. Detailed structural views of **2** at 100(2) K (*a*) and 300(2) K (*b*), including the detailed presentation of the cyanido-bridged {$Cd^{II}Au^{I}_2$} trimetallic molecules (two types, with S-pda and R-pda ligands, shown on left and right sides, respectively), being alternately arranged with supramolecular layers consisting of *cis*-*Λ*-[Cd^{II}(*μ*-NC)$_2$(*S*-pda)$_2$] or *cis*-*Δ*-[Cd^{II}(*μ*-NC)$_2$(*R*-pda)$_2$] complexes (left and right sides, respectively), the visualization of Au···Au metallophilic interaction pattern with depicted intermetallic distances, and the asymmetric unit with the labeling scheme for selected symmetrically independent atoms. Thermal ellipsoids for the asymmetric unit are presented at the 50% probability level. H-atoms in some views were omitted for clarity. Colors: green with various hues = Cd centers with amine ligands attached to them, orange with various hues = Au centers with cyanido ligands.

Table S5. Comparison of detailed crystal structure parameters of **2** at 100(2) and 300(2) K.

compound	**2**	
T / K	100(2)	300(2)
selected bond distances and angles in *cis*-[Cd1(*μ*-NC)$_2$(pda)$_2$] complexes / Å, °		
Cd1−N1	2.408(8)	2.405(8)
Cd1−N3	2.318(7)	2.309(6)
Cd1−N4	2.372(8)	2.367(8)
N1−Cd1−N1	96.5(4)	96.3(4)
N3−Cd1−N3	171.6(4)	171.5(4)
N4−Cd1−N4	98.5(4)	97.5(4)
N1−Cd1−N3	86.8(3)/98.8(3)	86.5(3)/99.2(3)
N1−Cd1−N4	85.4(3)/161.9(2)	86.1(3)/161.3(2)
N3−Cd1−N4	75.1(3)/99.3(3)	74.8(2)/99.5(3)
intermetallic distances and other structural parameters / Å, °		
Au1−Cd1	5.242(17)	5.224(17)
C1−N1−Cd1	140.8(10)	141.3(9)
Au1−Au2	3.221(17)	3.269(17)
hydrogen bond parameters (within a single aurophilic-interaction-based layer) / Å, °		
N1···N4<br>N1···H4A<br>N1···H4A−N4	3.32<br>2.50<br>155.0	3.41<br>2.58<br>154.3
N2···N3<br>N2···H3B<br>N2···H3B−N3	3.14<br>2.29<br>161.6	3.18<br>2.33<br>160.0
Au1···N4<br>Au1···H4B<br>Au1···H4B−N4	3.78<br>2.93<br>160.8	3.80<br>2.95<br>161.5
hydrogen bond parameters (between aurophilic-interaction-based layers) / Å, °		
N2···N3<br>N2···H3<br>N2···H3A−N3	3.04<br>2.22<br>154.0	3.08<br>2.27<br>151.7

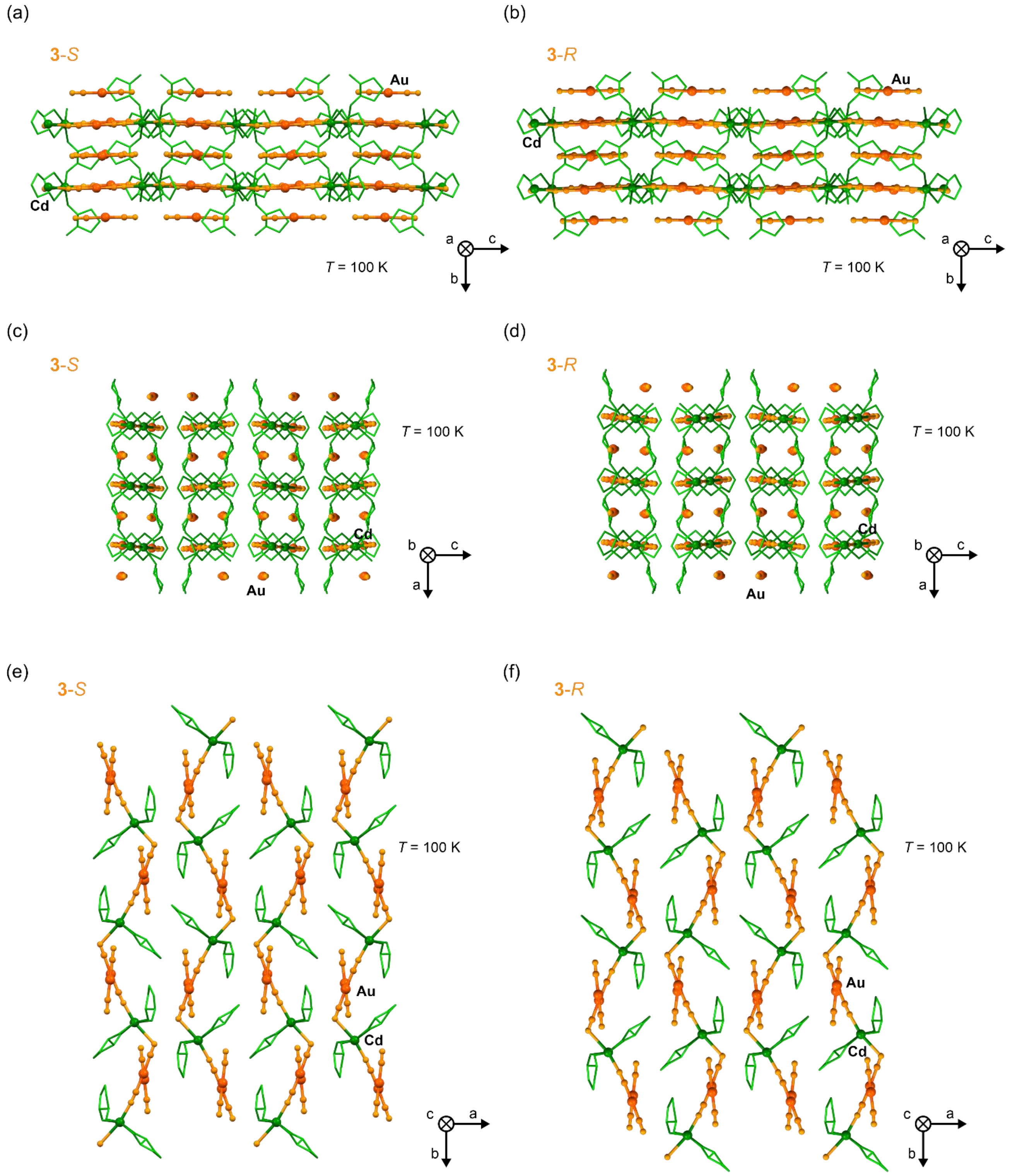

Fig. S9. The representative views of the coordination framework of **3**-*S* (left panel, i.e., *a*, *c*, and *e* parts) and **3**-*R* (right panel, i.e., *b*, *d*, and *f* parts) for the crystal structures determined at 100(2) K, presented along the main **a**, **b**, and **c** crystallographic axes (parts *a*–*b*, *c*–*d*, and *e*–*f*, respectively). Hydrogen atoms were omitted for clarity. Colors: green with various hues = Cd centers with amine ligands attached to them, orange with various hues = Au centers with cyanido ligands.

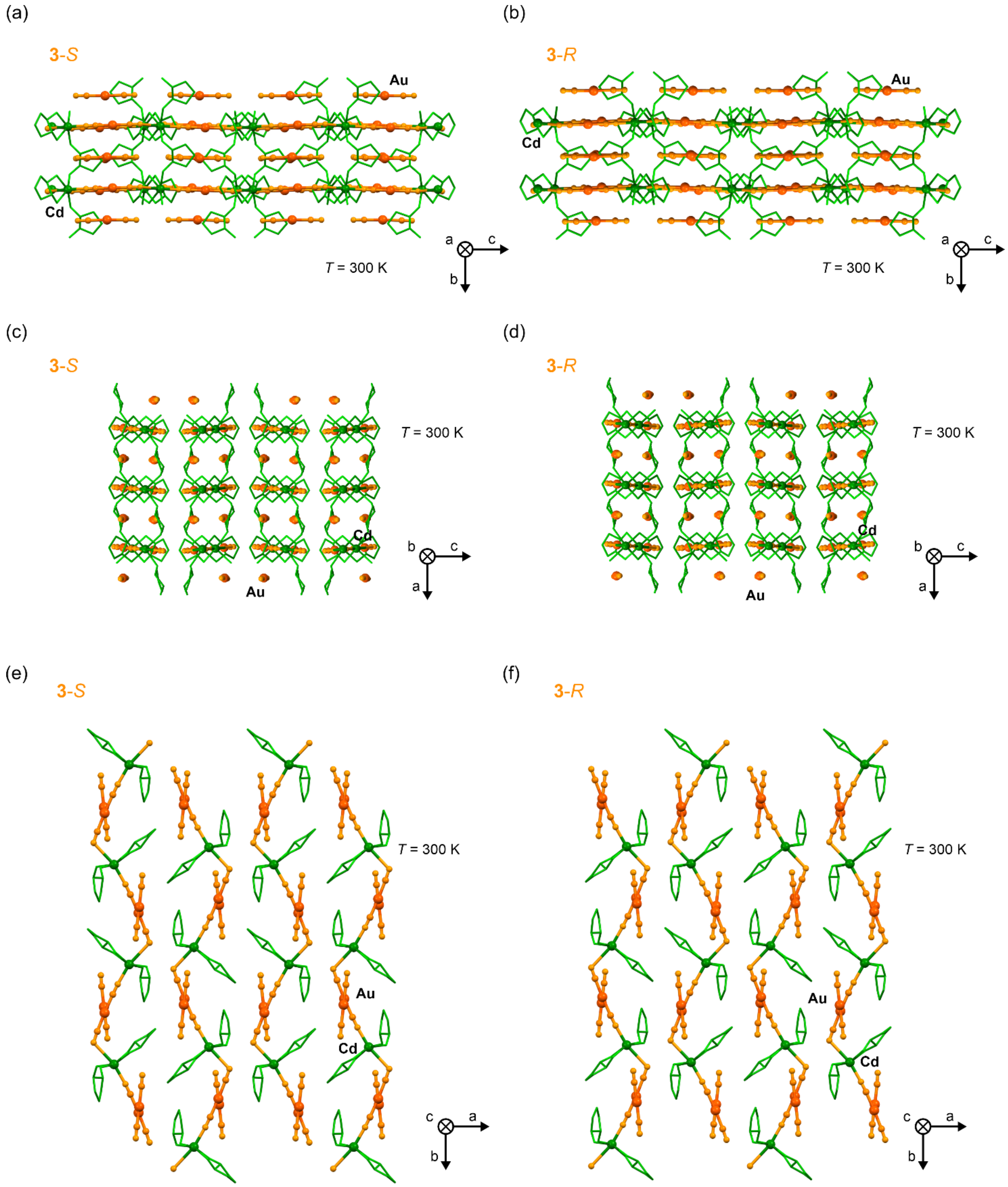

Fig. S10. The representative views of the coordination framework of **3**-*S* (left panel, i.e., *a*, *c*, and *e* parts) and **3**-*R* (right panel, i.e., *b*, *d*, and *f* parts) for the crystal structures determined at 300(2) K, presented along the main **a**, **b**, and **c** crystallographic axes (parts *a*–*b*, *c*–*d*, and *e*–*f*, respectively). Hydrogen atoms were omitted for clarity. Colors: green with various hues = Cd centers with amine ligands attached to them, orange with various hues = Au centers with cyanido ligands.

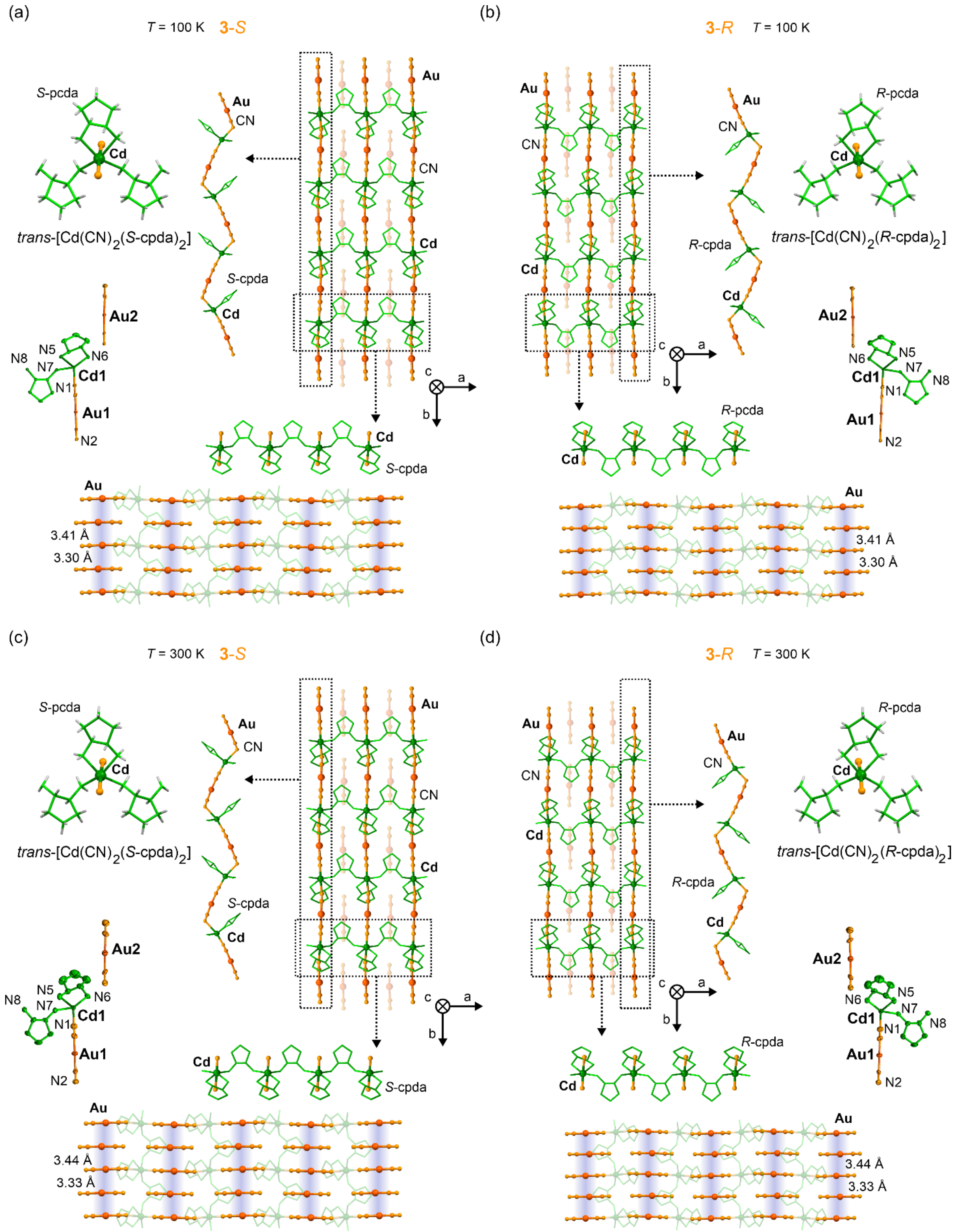

Fig. S11. Detailed structural views of **3-***S* (left panel, i.e., *a* and *c* parts) and **3-***R* (right panel, i.e., *b* and *d* parts) at 100(2) K (upper part, i.e., *a* and *b* parts) and 300(2) K (bottom part, i.e., *c* and *d* parts), including the detailed presentation of cyanido-bridged $\{Cd^{II}Au^{I}\}$-based mixed inorganic-organic layers (I^1O^1) with the demonstration of the metal-organic and inorganic connectivities within the layers, the visualization of Au···Au metallophilic interaction pattern with depicted intermetallic distances, and the asymmetric unit with the labeling scheme for selected symmetrically independent atoms. Thermal ellipsoids for the asymmetric unit are presented at the 50% probability level. Hydrogen atoms in some views were omitted for clarity. Colors: green with various hues = Cd centers with amine ligands attached to them, orange with various hues = Au centers with cyanido ligands.

Table S6. Comparison of detailed crystal structure parameters of **3**-*S* and **3**-*R* at 100(2) and 300(2) K.

compound	**3**-*S*		**3**-*R*	
T / K	100(2)	300(2)	100(2)	300(2)
selected bond distances and angles in *trans*-[Cd1(*μ*-NC)$_2$(*μ*-cpda)(cpda)] complexes / Å, o				
Cd1−N1	2.230(5)	2.225(8)	2.226(7)	2.233(10)
Cd1−N2	2.889(14)	2.955(17)	2.892(11)	2.938(22)
Cd1−N5	2.314(6)	2.334(9)	2.319(7)	2.317(9)
Cd1−N6	2.353(6)	2.363(11)	2.339(7)	2.347(11)
Cd1−N7	2.328(6)	2.361(10)	2.368(8)	2.325(10)
Cd1−N8	2.374(7)	2.323(8)	2.333(8)	2.382(12)
N1−Cd1−N2	166.97(3)	166.23(3)	166.74(3)	166.55(4)
N1−Cd1−N5	101.9(2)	101.9(4)	101.5(3)	101.7(4)
N1−Cd1−N6	104.8(2)	104.9(4)	105.3(3)	105.2(4)
N1−Cd1−N7	96.4(2)	97.2(4)	96.8(3)	96.7(5)
N1−Cd1−N8	97.2(2)	97.5(4)	96.9(3)	97.5(4)
N2−Cd1−N5	69.61(2)	69.03(4)	69.33(3)	69.29(4)
N2−Cd1−N6	67.32(2)	67.21(4)	67.44(3)	67.26(4)
N2−Cd1−N7	94.17(2)	94.12(4)	94.74(3)	93.91(4)
N2−Cd1−N8	92.47(2)	92.80(4)	92.42(3)	92.85(4)
N5−Cd1−N6	96.2(2)	97.0(3)	96.2(3)	97.1(4)
N5−Cd1−N7	157.2(2)	87.1(3)	87.7(3)	156.0(4)
N5−Cd1−N8	87.7(2)	156.2(4)	157.2(3)	87.4(4)
N6−Cd1−N7	156.2(2)	155.8(3)	156.2(3)	156.1(4)
N6−Cd1−N8	92.2(2)	91.9(3)	91.8(3)	91.5(4)
N7−Cd1−N8	76.40(18)	76.2(3)	76.7(2)	76.1(3)
intermetallic distances and other structural parameters / Å, º				
Au1−Cd1	5.135(14)/5.338(14)	5.147(17)/5.329(17)	5.138(11)/5.344(11)	5.143(22)/5.326(22)
C1−N1−Cd1	175.3(5)	176.8(9)	175.3(7)	175.9(11)
C2−N2−Cd1	117.85(8)	117.54(9)	117.66(7)	117.99(8)
Au1−Au2	3.295(14)/3.411(14)	3.329(17)/3.437(17)	3.297(11)/3.411(11)	3.326(22)/3.437(22)

hydrogen bond parameters (involving non-coordinating [Au(CN)$_2$]$^-$ ions) / Å, °				
N3···N5<br>N3···H5B<br>N3···H5B−N5	3.27<br>2.41<br>162.4	3.02<br>2.15<br>165.9	3.26<br>2.39<br>163.6	3.01<br>2.14<br>168.0
N3···N6<br>N3···H6B<br>N3···H6B−N6	3.38<br>2.49<br>155.0	3.40<br>2.60<br>149.7	3.39<br>2.55<br>154.8	3.40<br>2.63<br>141.7
N3···N7<br>N3···H7A<br>N3···H7A−N7	2.98<br>2.12<br>164.2	3.28<br>2.42<br>163.3	3.00<br>2.14<br>163.3	3.29<br>2.42<br>163.6
N3···N8<br>N3···H8B<br>N3···H8B−N8	3.23<br>2.44<br>148.5	3.26<br>2.46<br>149.2	3.23<br>2.14<br>147.8	3.26<br>2.47<br>148.5
N4···N7<br>N4···H7B<br>N4···H7B−N7	3.21<br>2.37<br>156.4	3.24<br>2.40<br>157.2	3.20<br>2.37<br>155.4	3.24<br>2.39<br>160.3
N4···N8<br>N4···H8A<br>N4···H8A−N8	3.13<br>2.25<br>170.0	3.15<br>2.27<br>171.1	3.14<br>2.26<br>169.3	3.17<br>2.28<br>171.4
hydrogen bond parameters (involving coordinating [Au(CN)$_2$]$^-$ ions) / Å, °				
N2···N5<br>N2···H5A<br>N2···H5B−N5	3.43<br>2.56<br>164.3	3.45<br>2.58<br>165.3	3.42<br>2.56<br>165.1	3.46<br>2.59<br>165.5
N2···N6<br>N2···H6A<br>N2···H6A−N6	3.16<br>2.31<br>163.9	3.20<br>2.30<br>177.2	3.15<br>2.26<br>170.7	3.21<br>2.32<br>170.4

Table S7. Results of Continuous Shape Measure (CShM) analyses for Cd(II) complexes in **1**-*S*, **1**-*R*, **2**, **3**-*S*, and **3**-*R*.

compound	complex	*T* / K	Continuous Shape Measure parameters*				
			HP-6	PPY-6	OC-6	TPR-6	JPPY-6
**1**-*S*	*cis*-[Cd1(*μ*-NC)$_2$(*S*-pda)$_2$]	100(2)	31.588	22.146	**2.559**	11.583	26.055
		300(2)	32.068	22.149	**2.635**	11.580	26.110
**1**-*R*	*cis*-[Cd1(*μ*-NC)$_2$(*R*-pda)$_2$]	100(2)	31.545	22.080	**2.544**	11.449	25.972
		300(2)	32.045	22.245	**2.611**	11.782	26.195
**2**	*cis*-[Cd1(*μ*-NC)$_2$(pda)$_2$]	100(2)	32.341	22.492	**2.120**	11.327	26.439
		300(2)	32.671	22.510	**2.194**	11.381	26.511
**3**-*S*	*trans*-[Cd1(*μ*-NC)$_2$ (*μ*-*S*-cpda)(*S*-cpda)]	100(2)	28.966	21.866	**2.664**	12.232	25.960
		300(2)	28.915	21.754	**2.800**	12.332	25.866
**3**-*R*	*trans*-[Cd1(*μ*-NC)$_2$ (*μ*-*R*-cpda)(*R*-cpda)]	100(2)	28.943	21.964	**2.610**	12.391	26.062
		300(2)	28.889	21.736	**2.838**	12.289	25.809

Table S8. Results of Continuous Shape Measure (CShM) analyses for Au(I) complexes in **1**-*S*, **1**-*R*, **2**, **3**-*S*, and **3**-*R*.

compound	complex	Continuous Shape Measure parameters*					
		100(2) K			300(2) K		
		L-2	vT-2	vOC-2	L-2	vT-2	vOC-2
**1**-*S*	coordinating $[Au1(\mu\text{-}CN)_2]^-$	**0.027**	13.148	23.581	**0.021**	13.001	23.396
	counter-ion $[Au2(\mu\text{-}CN)_2]^-$	**0.001**	14.058	24.718	**0.001**	14.135	24.813
**1**-*R*	coordinating $[Au1(\mu\text{-}CN)_2]^-$	**0.036**	12.976	23.365	**0.026**	13.171	23.611
	counter-ion $[Au2(\mu\text{-}CN)_2]^-$	**0.001**	14.018	24.668	**0.002**	13.989	24.632
**2**	coordinating $[Au1(\mu\text{-}CN)_2]^-$	**0.015**	13.447	23.957	**0.014**	13.471	23.987
**3**-*S*	coordinating $[Au1(\mu\text{-}CN)_2]^-$	**0.018**	13.360	23.848	**0.022**	13.289	23.758
	counter-ion $[Au2(\mu\text{-}CN)_2]^-$	**0.010**	13.606	24.154	**0.012**	13.660	24.221
**3**-*R*	coordinating $[Au1(\mu\text{-}CN)_2]^-$	**0.017**	13.392	23.888	**0.019**	13.353	23.839
	counter-ion $[Au2(\mu\text{-}CN)_2]^-$	**0.007**	13.854	24.463	**0.008**	13.745	24.328

Comment to Tables S7 and S8: The Continuous Shape Measure (CShM) parameter represents the distortion from ideal geometry. It equals 0 for an ideal polyhedron and increases with the increasing level of distortion. CShM Parameters for six-coordinated cadmium(II) complexes include HP-6 = parameter related to hexagon (D_{6h}), PPY-6 = pentagonal pyramid (C_{5v}), OC-6 = octahedron (O_h), TPR-6 = trigonal prism (D_{3h}), JPPY-6 = Johnson pentagonal pyramid (C_{5v}); for two-coordinated complexes, include L-2 = parameter related to line ($D_{\infty h}$), vT-2 = divacant tetrahedron (C_{2v}), vOC-2 = tetravacant octahedron (C_{2v}).[S4,S5]

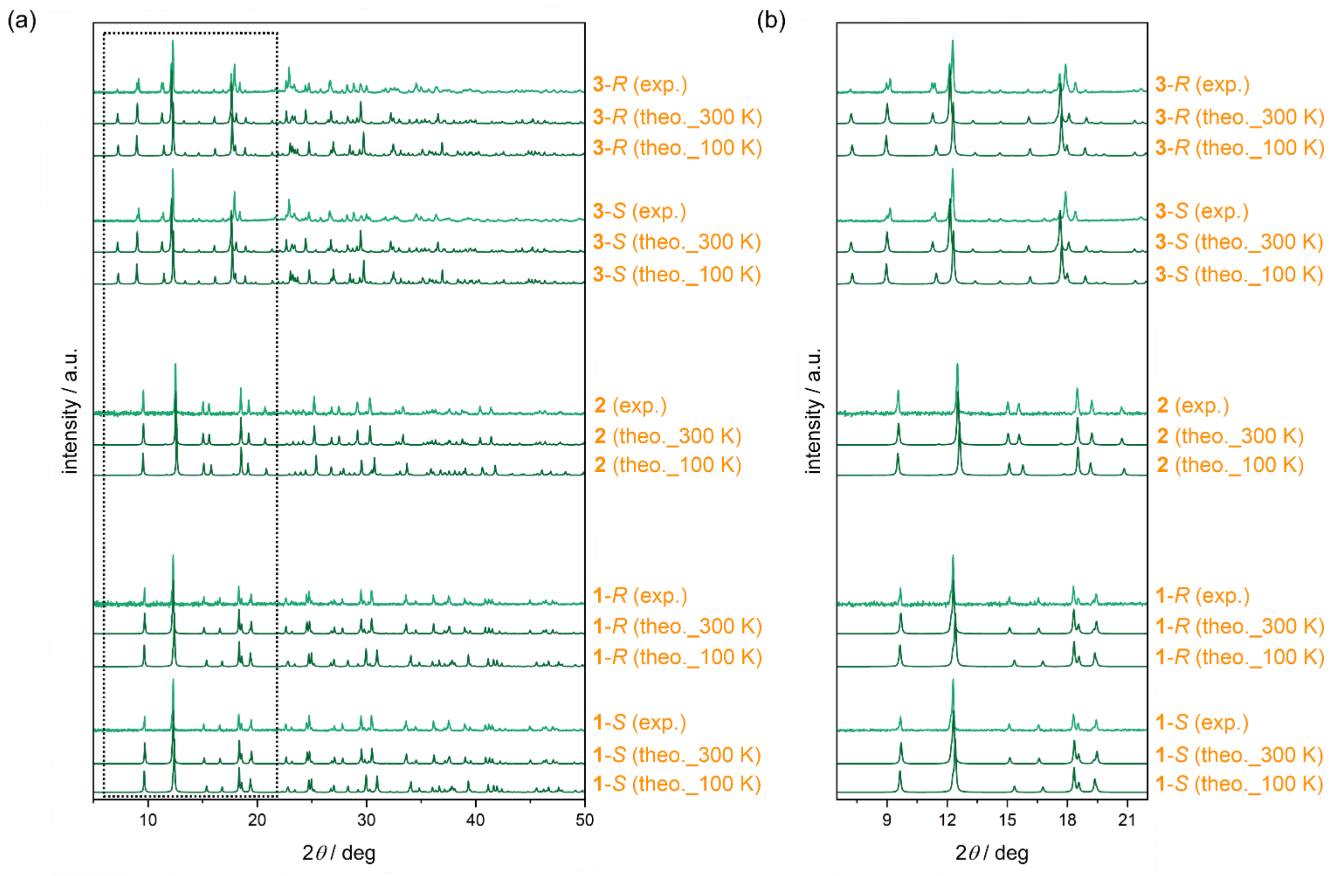

Fig. S12. Experimental (T = 300(2) K) and calculated powder X-ray diffraction patterns of **1**-*S*, **1**-*R*, **2**, **3**-*S*, and **3**-*R* presented in the broad 2Θ range of 5–50° (*a*) and the limited low-angle region of 6–22° (*b*). Experimental data were compared with the patterns calculated from the respective structural models obtained from the single-crystal X-ray diffraction (SC-XRD) structural analysis (T = 100(2) K and T = 300(2) K).

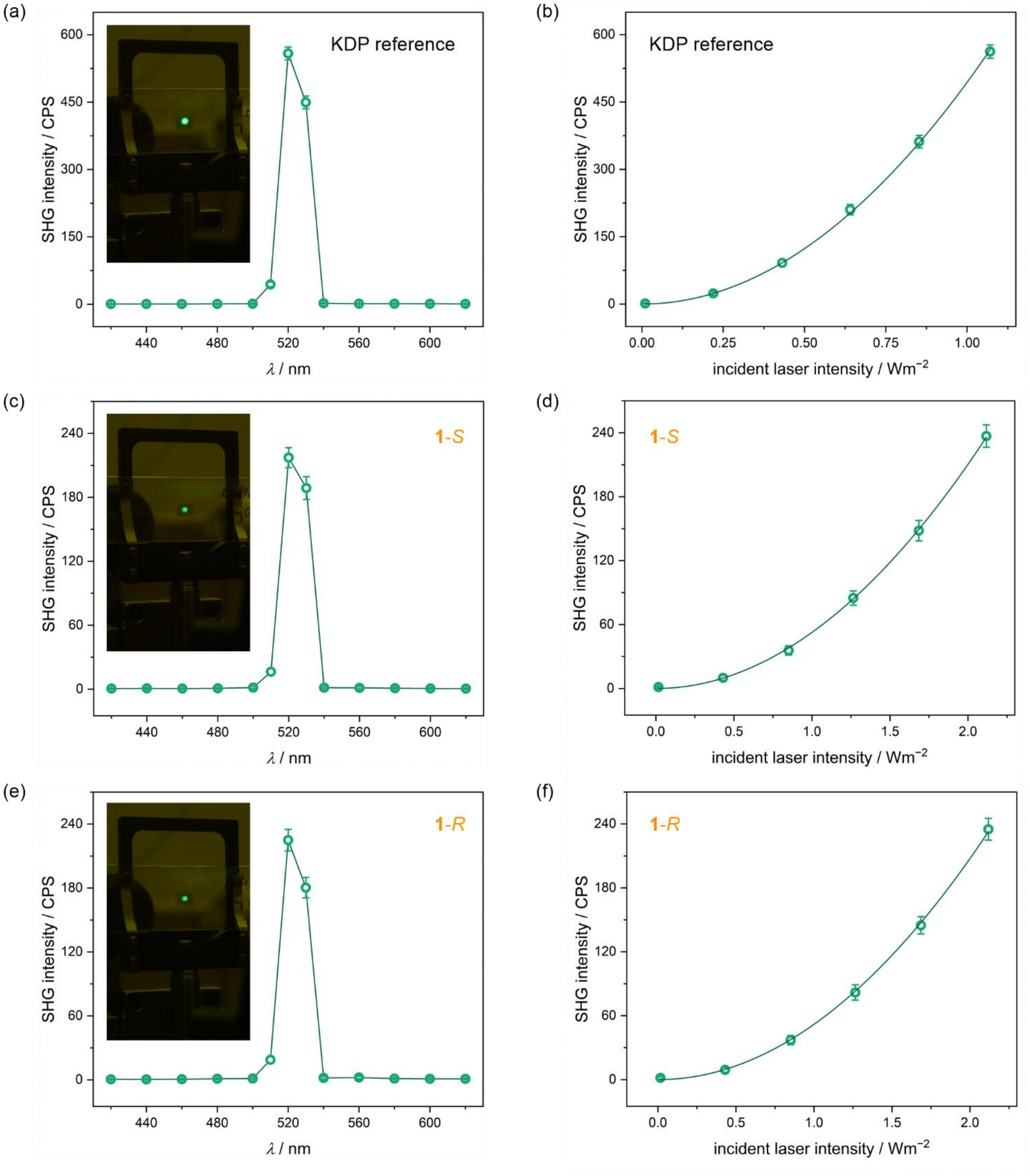

Fig. S13. The wavelength-dependences of the SHG signal (left panel, i.e., *a*, *c*, and *e* parts) and the dependences of the SHG light intensity on the excitation light intensity (right panel, i.e., *b*, *d*, and *f* parts) for the reference of potassium dihydrogen phosphate (**KDP**; *a*, *b*) and the indicated compounds **1**-*S* (*c*, *d*) and **1**-*R* (*e*, *f*). In the right panel, the coloured points represent the experimental results while the solid lines represent the best-fit curves according to the quadratic function indicating the second-harmonic nature of detected light. The resulting parameters are gathered in Table S9. Inside the left panel, the photos of the observed SHG light from the respective samples under the 1040 nm laser irradiation are also presented.

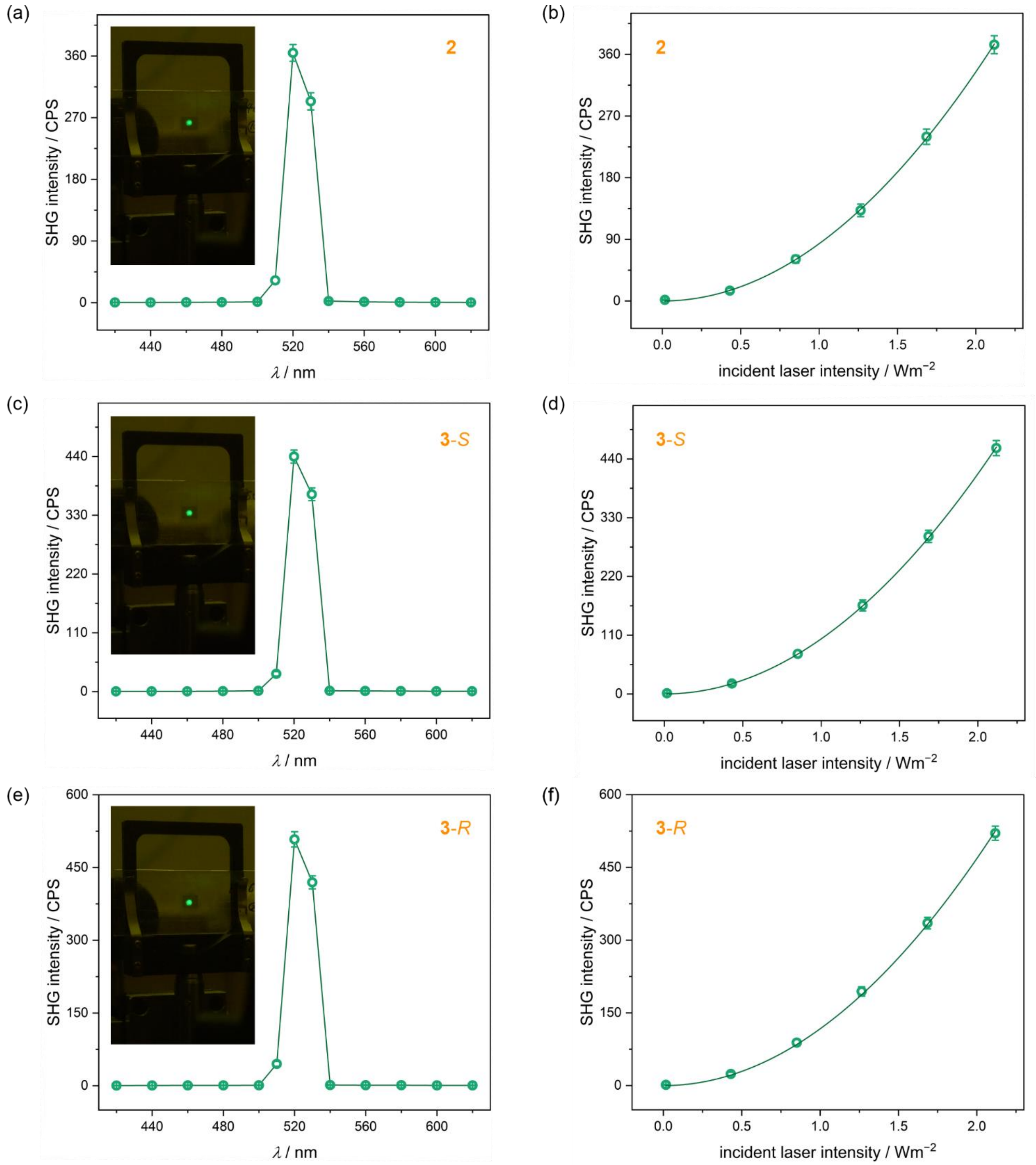

Fig. S14. The wavelength-dependences of the SHG signal (left panel, i.e., *a*, *c*, and *e* parts) and the dependences of the SHG light intensity on the excitation light intensity (right panel, i.e., *b*, *d*, and *f* parts) for the indicated compounds **2** (*a*, *b*), **3**-*S* (*c*, *d*), and **3**-*R* (*e*, *f*). In the right panel, the coloured points represent the experimental results while the solid lines represent the best-fit curves according to the quadratic function indicating the second-harmonic nature of detected light. The resulting parameters are gathered in Table S9. Inside the left panel, the photos of the observed SHG light from the respective samples under the 1040 nm laser irradiation are also presented.

Table S9. Comparison of the SHG intensities generated by the investigated samples of **1**-*S*, **1**-*R*, **2**, **3**-*S*, and **3**-*R* with the SH intensity generated by the potassium dihydrogen phosphate (KDP) reference sample (see Fig. S13 and S14 for detailed characteristics).

material	quadratic term coefficient (±error)	percentage of KDP / %
KDP - reference	493.90 (±2.88)	100
**1**-*S*	52.61 (±0.26)	10.65 (±0.05)
**1**-*R*	51.90 (±0.32)	10.51 (±0.07)
**2**	83.61 (±0.27)	16.93 (±0.05)
**3**-*S*	103.01 (±0.27)	20.86 (±0.05)
**3**-*R*	117.09 (±0.78)	23.71 (±0.16)

Table S10. Results of dipole moment calculations of Cd(II) complexes in the crystal structures of **1**-*S*, **1**-*R*, **2**, **3**-*S*, and **3**-*R* measured at 300(2) K. Details of the calculations are described in the comment below. The resulting direction of the dipole moment vectors is presented in Fig. S15.

comp.	atom	Cd−N distance / Å	bond atomic valences	C_{charge}	$C_{gravity}$	μ / D
**1**-*S*	Cd	-	1.974	-	-	-
	N (cyanido)	2.442	−0.265	0.333	0.311	5.658
	N (amine)	2.293	−0.397	0.317	0.292	6.577
	N (amine)	2.367	−0.325	0.325	0,301	6.076
	**The resulting dipole moment is oriented along the *b* crystal. direction with a magnitude of 1.204(±0.028) D**					
**1**-*R*	Cd	-	1.925	-	-	-
	N (cyanido)	2.449	−0.260	0.333	0.312	5.550
	N (amine)	2.304	−0.385	0.318	0.293	6.427
	N (amine)	2.376	−0.317	0.326	0.302	5.950
	**The resulting dipole moment is oriented along the −*b* crystal. direction with a magnitude of 1.119(±0.029) D**					
**2**	Cd	-	1.994	-	-	-
	N (cyanido)	2.406	−0.292	0.329	0.306	5.878
	N (amine)	2.309	−0.380	0.319	0.294	6.489
	N (amine)	2.367	−0.325	0.325	0.301	6.105
	**The resulting dipole moment is oriented along the *c* crystal. direction with a magnitude of 1.168(±0.023) D**					
**3**-*S*	Cd	-	1.921	-	-	-
	N (cyanido)	2.225	−0.477	0.311	0.283	7.056
	N (cyanido)	2.955	−0.066	0.393	0.376	4.289
	N (bridging amine)	2.324	−0.365	0.320	0.296	6.280
	N (bridging amine)	2.362	−0.329	0.324	0.301	6.030
	N (chelate amine)	2.334	−0.355	0.321	0.297	6.212
	N (chelate amine)	2.363	−0.328	0.324	0.301	6.024
	**The resulting dipole moment has a magnitude of 2.562(±0.055) D** (orientation presented in Fig. S15)					
**3**-*R*	Cd	-	1.926	-	-	-
	N (cyanido)	2.233	−0.467	0.311	0.284	6.992
	N (cyanido)	2.938	−0.069	0.391	0.374	4.311
	N (bridging amine)	2.317	−0.372	0.320	0.295	6.335
	N (bridging amine)	2.348	−0.342	0.323	0.299	6.126
	N (chelate amine)	2.325	−0.364	0.320	0.296	6.280
	N (chelate amine)	2.382	−0.312	0.326	0.303	5.915
	**The resulting dipole moment has a magnitude of 2.419(±0.055) D** (orientation presented in Fig. S15)					

Table S11. Results of dipole moment calculations of Au(I) complexes in the crystal structures of **1**-*S*, **1**-*R*, **2**, **3**-*S*, and **3**-*R* measured at 300(2) K. Details of the calculations are described in the comment below. The resulting direction of the dipole moment vectors is presented in Fig. S15.

<table>
<tr><th>comp.</th><th>atom</th><th>Au−C distance / Å</th><th>bond atomic valences</th><th>C_{charge}</th><th>$C_{gravity}$</th><th>μ / D</th></tr>
<tr><td rowspan="8">1-S</td><td>Au (coordinating metalloligand)</td><td>-</td><td>1.236</td><td>-</td><td>-</td><td>-</td></tr>
<tr><td>C (cyanido)</td><td>1.982</td><td>−0.618</td><td>0.155</td><td>0.140</td><td>6.283</td></tr>
<tr><td>C (cyanido)</td><td>1.982</td><td>−0.618</td><td>0.155</td><td>0.140</td><td>6.283</td></tr>
<tr><td colspan="6">The resulting dipole moment is oriented along the −b crystal. direction with a magnitude of 0.283(±0.009) D</td></tr>
<tr><td>Au (counter-ion)</td><td>-</td><td>1.223</td><td>-</td><td>-</td><td>-</td></tr>
<tr><td>C (cyanido)</td><td>1.981</td><td>−0.611</td><td>0.155</td><td>0.140</td><td>6.228</td></tr>
<tr><td>C (cyanido)</td><td>1.981</td><td>−0.611</td><td>0.155</td><td>0.140</td><td>6.228</td></tr>
<tr><td colspan="6">The resulting dipole moment is oriented along the b crystal. direction with a magnitude of 0.039(±0.001) D</td></tr>
<tr><td rowspan="8">1-R</td><td>Au (coordinating metalloligand)</td><td>-</td><td>1.184</td><td>-</td><td>-</td><td>-</td></tr>
<tr><td>C (cyanido)</td><td>1.999</td><td>−0.592</td><td>0.156</td><td>0,141</td><td>6.066</td></tr>
<tr><td>C (cyanido)</td><td>1.999</td><td>−0.592</td><td>0.156</td><td>0.141</td><td>6.066</td></tr>
<tr><td colspan="6">The resulting dipole moment is oriented along the b crystal. direction with a magnitude of 0.295(±0.010) D</td></tr>
<tr><td>Au (counter-ion)</td><td>-</td><td>1.092</td><td>-</td><td>-</td><td>-</td></tr>
<tr><td>C (cyanido)</td><td>2.02</td><td>−0.546</td><td>0.157</td><td>0.143</td><td>5.677</td></tr>
<tr><td>C (cyanido)</td><td>2.02</td><td>−0.546</td><td>0.157</td><td>0.143</td><td>5.677</td></tr>
<tr><td colspan="6">The resulting dipole moment is oriented along the −b crystal. direction with a magnitude of 0.063(±0.004) D</td></tr>
<tr><td rowspan="4">2</td><td>Au (coordinating metalloligand)</td><td>-</td><td>1.214</td><td>-</td><td>-</td><td>-</td></tr>
<tr><td>C (cyanido)</td><td>1.978</td><td>−0.616</td><td>0.155</td><td>0.140</td><td>6.257</td></tr>
<tr><td>C (cyanido)</td><td>1.991</td><td>−0.598</td><td>0.156</td><td>0.141</td><td>6.131</td></tr>
<tr><td colspan="6">The resulting dipole moment is oriented along the c crystal. direction with a magnitude of 0.315(±0.012) D</td></tr>
<tr><td rowspan="8">3-S</td><td>Au (coordinating metalloligand)</td><td>-</td><td>1.233</td><td>-</td><td>-</td><td>-</td></tr>
<tr><td>C (cyanido)</td><td>1.988</td><td>−0.600</td><td>0.156</td><td>0.140</td><td>6.155</td></tr>
<tr><td>C (cyanido)</td><td>1.970</td><td>−0.633</td><td>0.155</td><td>0.139</td><td>6.386</td></tr>
<tr><td colspan="6">The resulting dipole moment has a magnitude of 0.489(±0.005) D</td></tr>
<tr><td>Au (counter-ion)</td><td>-</td><td>1.206</td><td>-</td><td>-</td><td>-</td></tr>
<tr><td>C (cyanido)</td><td>1.972</td><td>−0.630</td><td>0.155</td><td>0.139</td><td>6.344</td></tr>
<tr><td>C (cyanido)</td><td>2.005</td><td>−0.576</td><td>0.156</td><td>0.142</td><td>5.970</td></tr>
<tr><td colspan="6">The resulting dipole moment has a magnitude of 0.414(±0.012) D</td></tr>
</table>

**3**-*R*	Au (coordinating metalloligand)	-	1.262	-	-	-
	C (cyanide)	1.983	−0.610	0.155	0.140	6.243
	C (cyanide)	1.958	−0.652	0.154	0.138	6.537
	**The resulting dipole moment has a magnitude of 0.539(±0.012) D**					
	Au (counter-ion)	-	1.157	-	-	-
	C (cyanide)	1.983	−0.611	0.155	0.140	6.184
	C (cyanide)	2.023	−0.546	0.157	0.143	5.722
	**The resulting dipole moment has a magnitude of 0.478(±0.005) D**					

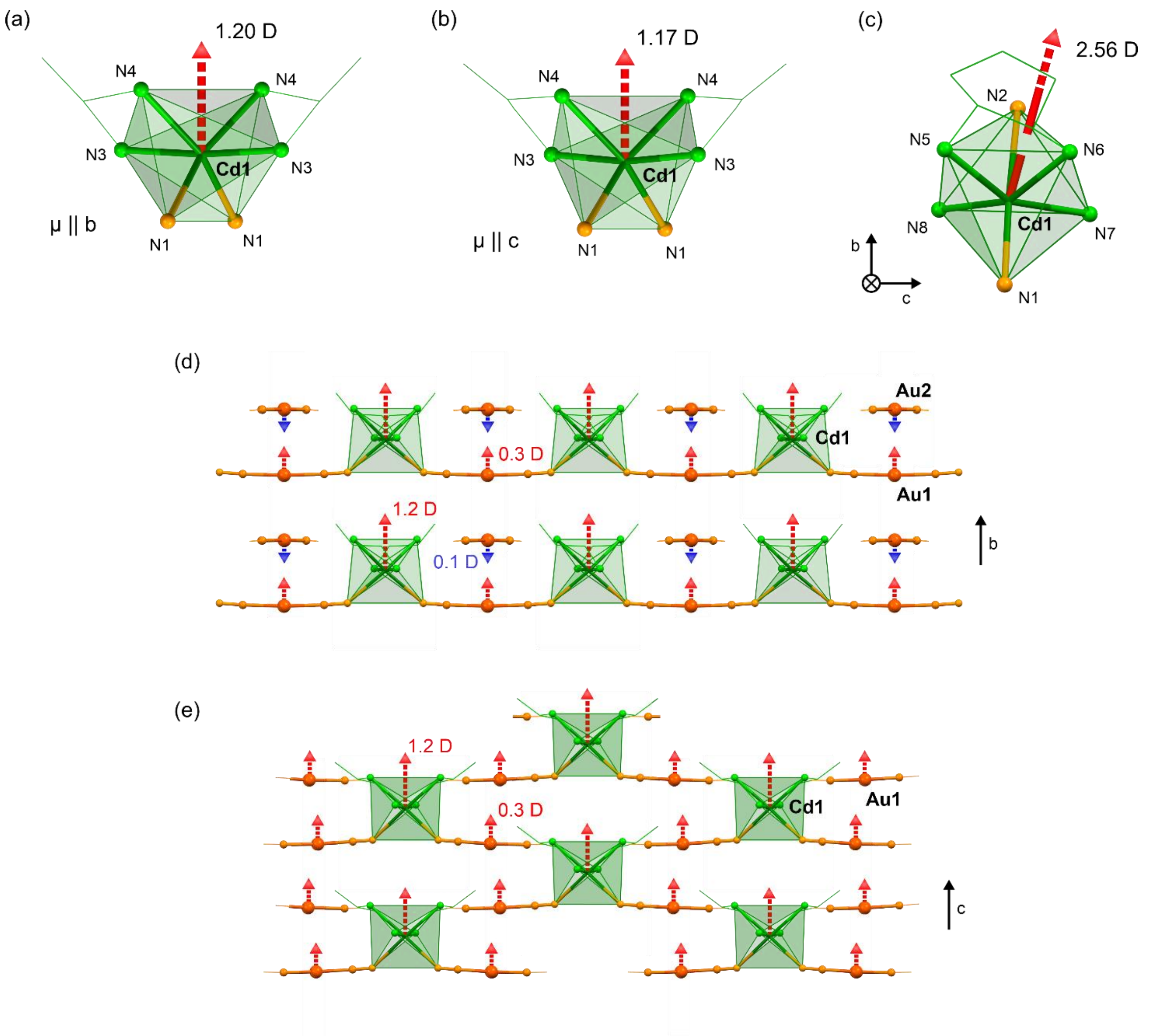

Fig. S15. The directions of dipole moment vectors (red arrows) in the {CdN_6} distorted octahedrons (Table S10) in the crystal structures of **1**-*S* (*a*), **2** (*b*), and **3**-*S* (*c*), and the schematic presentation of the directions of dipole moment vectors of all metal complexes as well as favorable and unfavorable dipole-dipole interactions (Tables S10 and S11) in the crystal structures of **1**-*S* (*d*) and **2** (*e*).

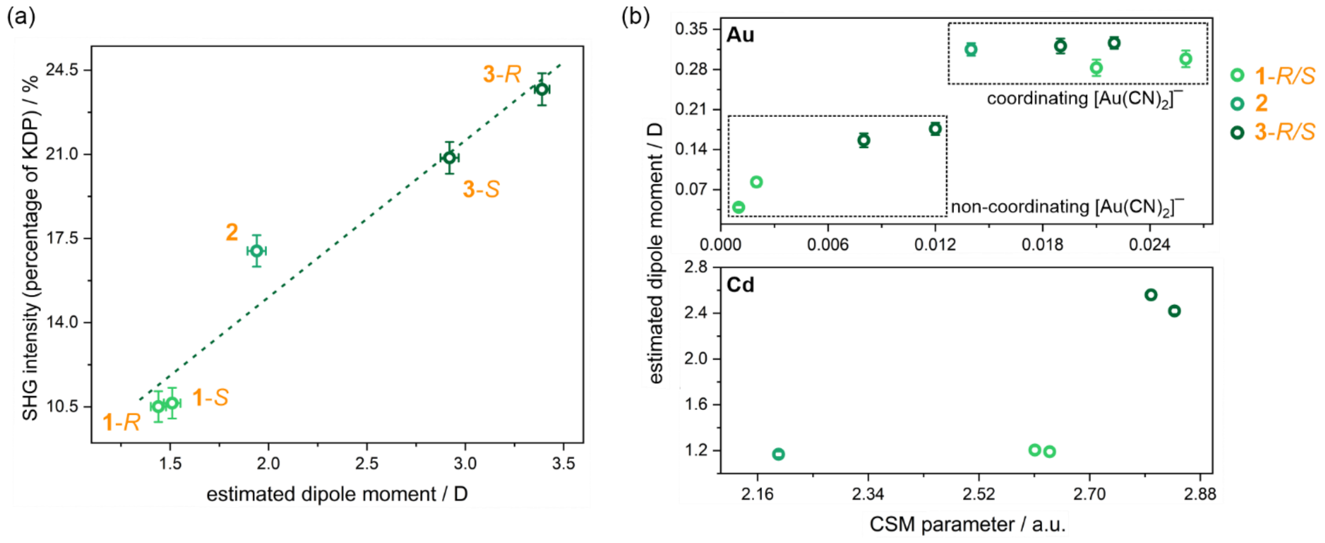

Fig. S16. The dependence of SHG intensities generated by the investigated samples of **1**-*S*, **1**-*R*, **2**, **3**-*S*, and **3**-*R* as a percentage of the KDP reference sample on the estimated dipole moment calculated for the {$CdAu_2$} unit (*a*), and the comparison of estimated dipole moment and the results of Continuous Shape Measure analyses for Cd(II) and Au(I) complexes in **1**-*S*, **1-***R*, **2**, **3**-*S*, and **3**-*R* materials (Tables S7−S11) (*b*).

Comment to Fig. S15 and S16, Tables S10 and S11 – dipole moment calculations

A simple bond-valence approach has been used to calculate the direction and magnitude of the dipole moment of complexes of all crystallographically independent ions, taking into account their non-ideal polyhedra (using data from the SC-XRD experiments at 300(2) K). The Debye equation, $\mu = n \cdot e \cdot R$ (μ is the net dipole moment, n is the total number of electrons, e is the charge on an electron, and R is the difference between "positive" and "negative" charge, $C_{gravity}$ and C_{charge}, respectively), was used to calculate the dipole moment of individual Cd−N and Au−C bonds, while the distribution of electrons on Cd, N, Au, and C atoms was estimated using bond-valence theory, $S_i = \exp[(R_0 - R_i)/B]$ (where R_0 and B are an empirical constant, R_i is the bond length).[S6–S8] The uncertainties were calculated using the total differential of the cumulative functions above.

For the Cd(II) coordination environment in **1**-*R*, $\{CdN_6\}$, the atomic valences were calculated to be Cd(+1.93), $N1_{NC}$(−0.26), $N3_{amine}$ (−0.39), and $N4_{amine}$ (−0.32), to give dipole moments of 5.6, 6.4 and 6.0 debye for Cd1−N1, Cd1−N3, and Cd1−N4, respectively. A vector sum of the dipole moments for all six Cd–N bonds gives a net dipole moment of 1.1 debye, directed along the **b** crystallographic axis towards the plane formed by the four coordinating nitrogen atoms of the pda amine ligands (Fig. S15, Table S10). Almost identical results were calculated for $\{CdN_6\}$ polyhedra in **1**-*S*. The calculations for similar Cd(II) complexes in **2** give the atomic valences Cd(+1.99), $N1_{NC}$(−0.29), $N3_{amine}$ (−0.38), and $N4_{amine}$ (−0.32), which results in similar dipole moments of 5.9, 6.5 and 6.1 debye for Cd1−N1, Cd1−N3, and Cd1−N4, respectively. The resultant vector sum of the dipole moments is directed toward the **c** crystallographic direction with the magnitude of 1.2 debye (Table S10). An identical calculation for the Cd(II) complexes in **3**-*S* and **3**-*R* gives the calculated dipole moments values of 7.1 and 4.3 debye for Cd−N of linear and bent cyanido molecular bridge, respectively, and 6.3−6.0 debye for remaining Cd−N bonds in **3**-*S*, as well as 7.0, 4.3, and 6.3−5.9 debye for respective values in **3**-*R*. For both analogs, the vector summation gives a net dipole moment of ca. 2.5 debye, larger than for Cd^{II} complexes in **1**-*S*, **1**-*R*, and **2**, directed roughly along the Cd1−N3 bond axis towards the bent cyanido bridge (Fig. S15, Table S10). The polyhedra of $[Au^{I}(CN)_2]^-$ complexes were also calculated. In **1**-*S*, **1**-*R*, and **2**, the magnitude of the vector sum of the dipole moments is ca. 0.3 debye for coordinated metalloligands, while for non-coordinated complexes it is less than 0.07 debye (Table S11). Respective calculations in **3**-*S* and **3**-*R* give values of 0.41−0.54 debye (Table S11). The applied method of estimating the dipole moments is error-prone as it does not take into account the correct distribution of electrons within the complexes, affected by ionic interactions and the formation of hydrogen bonds by cyanido ligands and N−H amine groups (see the crystal structure description), however, it reflects a certain trend and allows to explain the differences in non-linear optical properties of compounds (Fig. S16).[S6–S8]

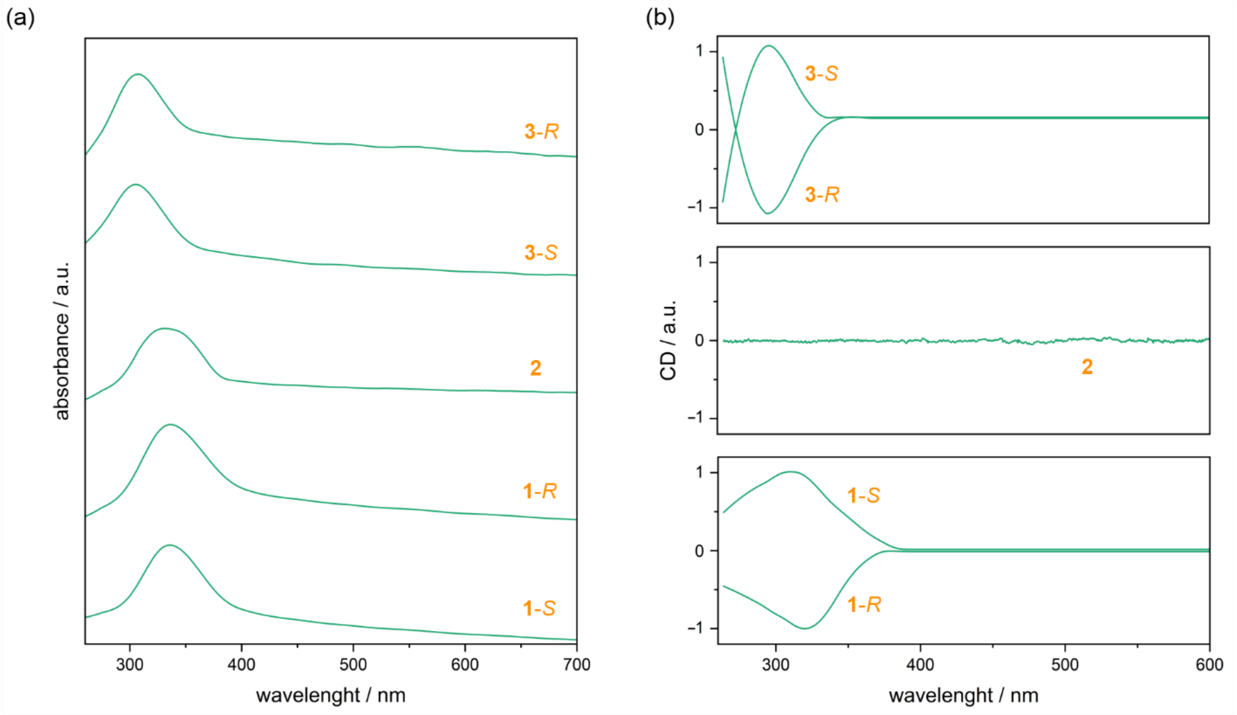

Fig. S17. Solid-state UV-vis absorption spectra (a) and circular dichroism (CD) spectra (b) for **1**-*S*, **1**-*R*, **2**, **3**-*S*, and **3**-*R* materials presented in the 260−700 and 260−600 nm range, respectively.

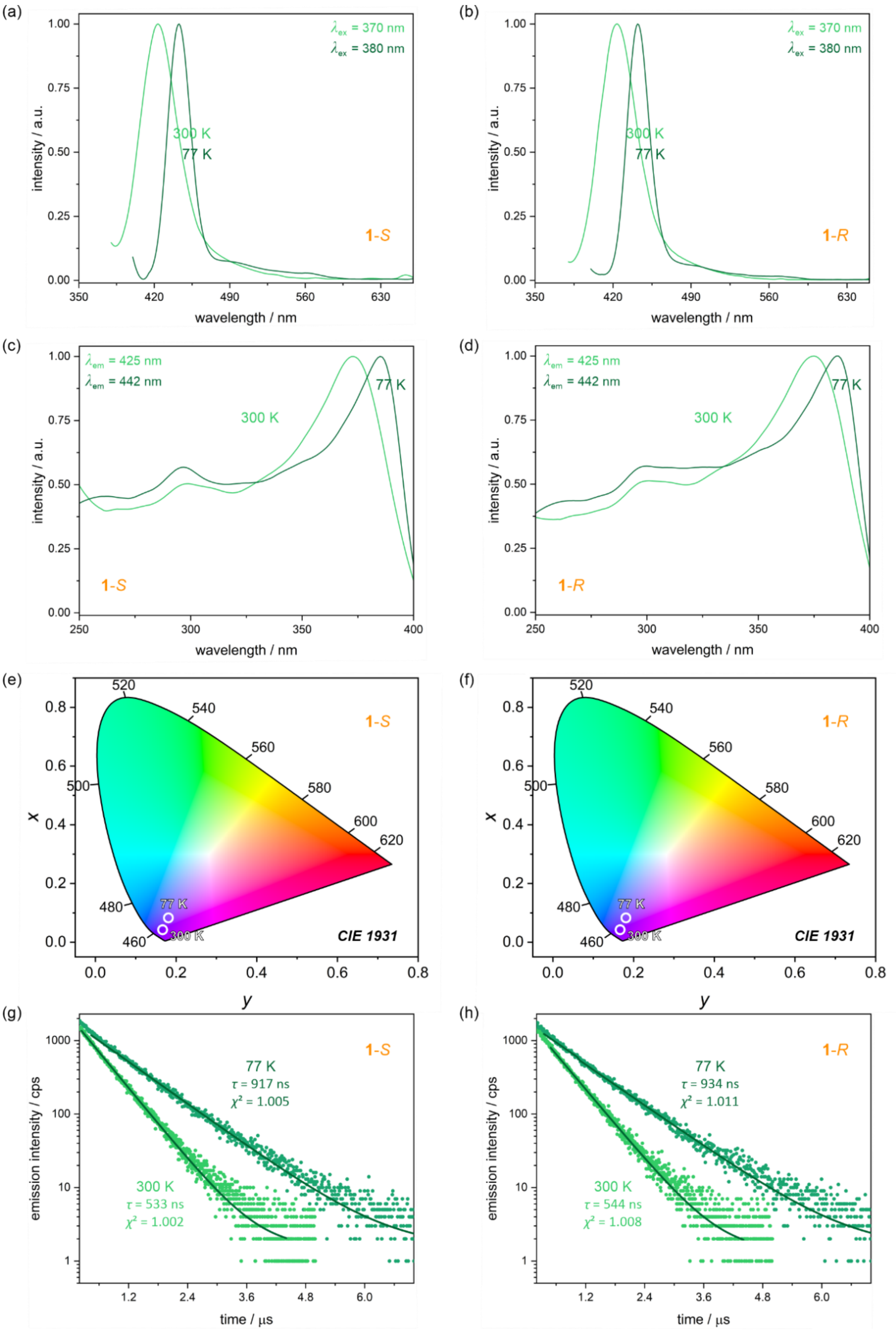

Fig. S18. Selected curves of solid-state photoluminescent properties of **1**-*S* (left panel, i.e., *a*, *c*, *e*, and *g* parts) and **1**-*R* (right panel, i.e., *b*, *d*, *f*, and *h* parts), including the comparison of low- and high-temperature (77 and 300 K, respectively) emission (*a*, *b*) and excitation (*c*, *d*) spectra collected under indicated wavelengths, emission colors presented on the CIE 1931 chromaticity diagram (*e*, *f*), and comparison of low- and high-temperature (77 and 300 K, respectively) emission decay profiles (*g*, *h*). The best-fit parameters, as well as the CIE 1931 chromaticity parameters, are gathered in Table S12.

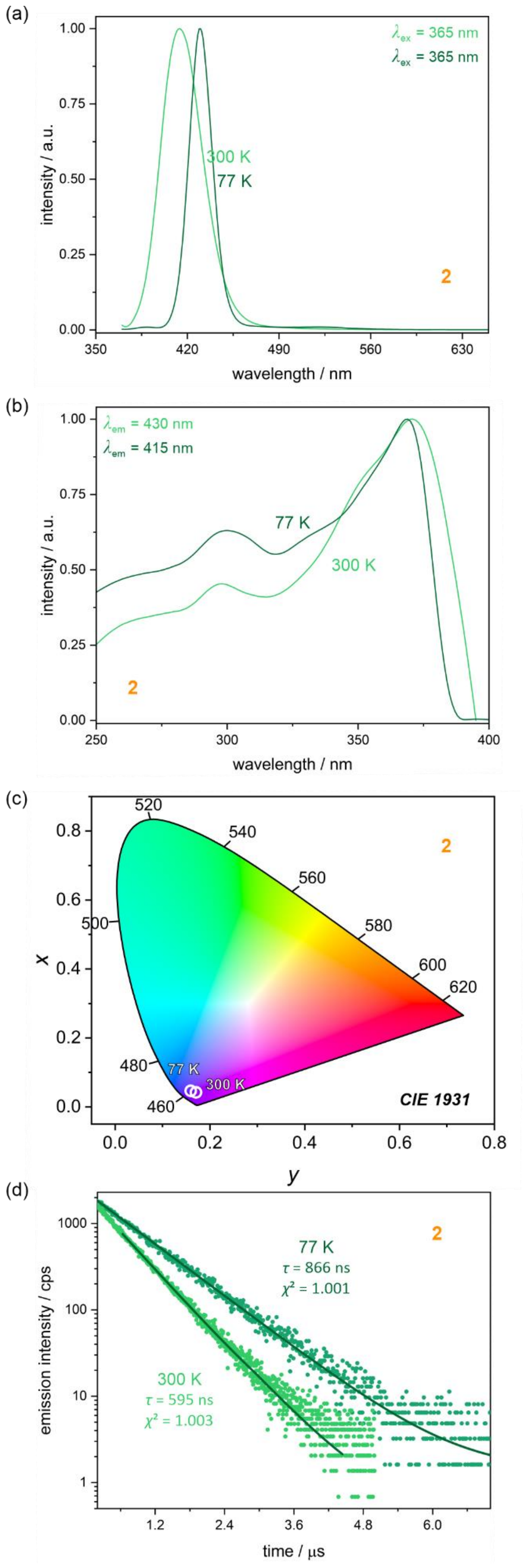

Fig. S19. Selected curves of solid-state photoluminescent properties of **2**, including the comparison of low- and high-temperature (77 and 300 K, respectively) emission (*a*) and excitation (*b*) spectra collected under indicated wavelengths, emission colors presented on the CIE 1931 chromaticity diagram (*c*), and comparison of low- and high-temperature (77 and 300 K, respectively) emission decay profiles (*d*). The best-fit parameters, as well as the CIE 1931 chromaticity parameters, are gathered in Table S12.

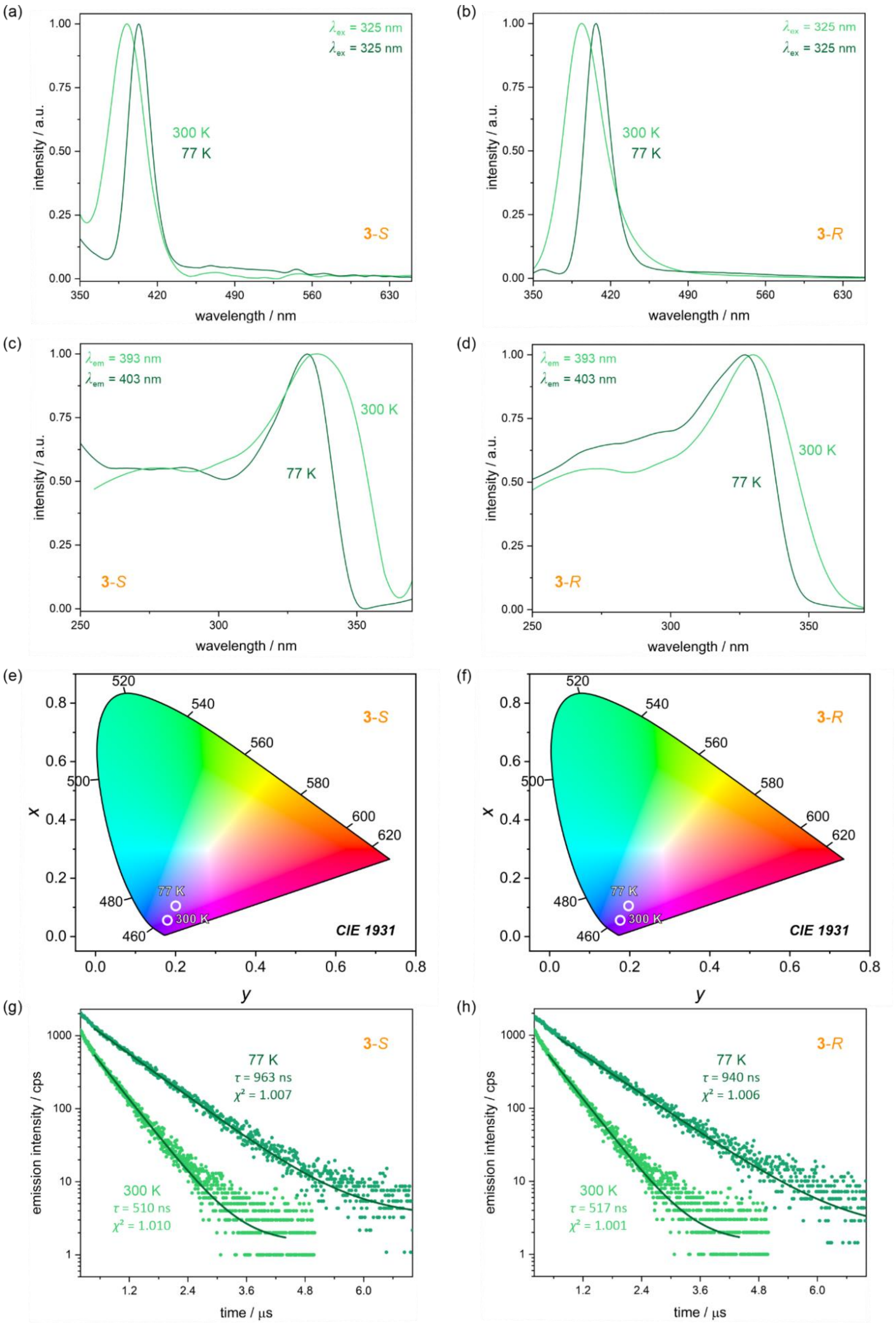

Fig. S20. Selected curves of solid-state photoluminescent properties of **3**-*S* (left panel, i.e., *a*, *c*, *e*, and *g* parts) and **3**-*R* (right panel, i.e., *b*, *d*, *f*, and *h* parts), including the comparison of low- and high-temperature (77 and 300 K, respectively) emission (*a*, *b*) and excitation (*c*, *d*) spectra collected under indicated wavelengths, emission colors presented on the CIE 1931 chromaticity diagram (*e*, *f*), and comparison of low- and high-temperature (77 and 300 K, respectively) emission decay profiles (*g*, *h*). The best-fit parameters, as well as the CIE 1931 chromaticity parameters, are gathered in Table S12.

Table S12. Selected spectroscopic parameters of the solid-state photoluminescent properties of **1**-*S*, **1**-*R*, **2**, **3**-*S*, and **3**-*R* at 77 and 300 K (Fig. S18−S20), including the position of an emission pattern maximum, the CIE 1931 chromaticity parameters, the best-fit parameters for the emission decay profiles to the mono-exponential function, and the absolute quantum yield.

compound	T / K	λ_{max} / nm	CIE 1931 chromaticity parameters		best-fit parameters for the emission decay profiles		absolute quantum yield, φ_{em}
			x	y	τ ($\pm\Delta\tau$) / ns	χ^2	
**1**-*S*	77(2)	442	0.166	0.063	917 (±4)	1.005	−
	300(2)	425	0.166	0.043	533 (±2)	1.002	0.73
**1**-*R*	77(2)	442	0.167	0.068	934 (±5)	1.011	−
	300(2)	425	0.163	0.0370	544 (±2)	1.008	0.74
**2**	77(2)	430	0.168	0.022	866 (±3)	1.001	−
	77(2)	415	0.167	0.016	595 (±4)	1.003	0.83
**3**-*S*	77(2)	403	0.189	0.093	963 (±4)	1.007	−
	300(2)	393	0.170	0.054	510 (±3)	1.010	0.39
**3**-*R*	77(2)	403	0.188	0.090	940 (±3)	1.006	−
	300(2)	393	0.169	0.059	517 (±2)	1.001	0.42

Additional comment on the observed values of emission lifetimes and their relation to the emission mechanism. One can consider a thermally activated delayed fluorescence (TADF) as a potential luminescence mechanism in the reported systems. To investigate whether the radiative process in our systems is phosphorescence or a TADF process, we studied the temperature dependence of the decay. In the TADF process, the delayed fluorescence is heavily suppressed when the sample is cooled, indicating that the delayed component arises from a thermally activated process. On the other hand, the long-lived phosphorescence shows the opposite trend, being present at low temperatures but suppressed when the sample is warmed to RT. In the submitted work, we measured the luminescence lifetime at room (300 K) and liquid nitrogen (77 K) temperature. As presented in Table S12 above, with increasing temperature, the lifetime decreased twofold, which rather indicates phosphorescence as an emission mechanism. Moreover, theoretical calculations and experimental investigations on similar types of compounds containing aurophilic interactions support the interpretation of the observed emission from the triplet excited state (please see the section **Photoluminescence and other optical properties** of the main article).

Table S13. Comparison of photoluminescence lifetime, absolute quantum yield, and the radiative and nonradiative decay rate constants of **1**-*S*, **1**-*R*, **2**, **3**-*S*, and **3**-*R* at 300(2) K.

compound	τ ($\pm\Delta\tau$) / ns	absolute quantum yield, φ_{em} / %	decay rate constant / 10^5 s^{-1}	
			radiative, k_r	nonradiative, k_{nr}
**1**-*S*	533 (±2)	72.79(3)	13.66 (±0.051)	0.50 (±0.002)
**1**-*R*	544 (±2)	73.89(5)	13.58 (±0.050)	0.48 (±0.002)
**2**	595 (±4)	83.45(2)	14.03 (±0.094)	0.28 (±0.002)
**3**-*S*	510 (±3)	39.25(8)	7.70 (±0.045)	1.19 (±0.007)
**3**-*R*	517 (±2)	41.80(1)	8.09 (±0.031)	1.13 (±0.004)

Comment to Table S13: the radiative (k_r) and nonradiative (k_{nr}) decay rate constants are based on the relationship $k_r = \varphi_{em}/\tau$ and $\varphi_{em} = k_r/(k_r+k_{nr})$ in which τ values are luminescence lifetimes and the φ_{em} is the related absolute quantum yield.[S9,S10]

Literature to the Supporting Information:

[S1]	K. Kumar, O. Stefańczyk, S. Chorazy, K. Nakabayashi, B. Sieklucka and S. Ohkoshi, *Inorg. Chem*., 2019, **58**, 5677−5687.
[S2]	J. Lefebvre, J. L. Korčok, M. J. Katz and D. B. Leznoff, *Sensors*, 2012, **12**, 3669−3692.
[S3]	A. Yamagishi, T. Kawasaki, K. Hiruma, H. Sato and T. Kitazawa, *Dalton Trans.*, 2016, **45**, 7823−7828.
[S4]	M. Llunell, D. Casanova, J. Cirera, J. Bofill, P. Alemany and S. Alvarez, M. Pinsky, D. Avnir SHAPE v. 2.1. University of Barcelona: Barcelona, Spain, 2013.
[S5]	D. Casanova, J. Cirera, M. Llunell, P. Alemany, D. Avnir and S. Alvarez, *J. Am. Chem. Soc.*, 2004, **126**, 1755−1763.
[S6]	P. A. Maggard, T. S. Nault, C. L. Stern and K. R. Poeppelmeier, *J. Solid State Chem*., 2003, **175**, 27−33.
[S7]	D. Altermatt and I. D. Brown, *Acta Cryst.*, 1985, **B41**, 240−244.
[S8]	Y. J. Kim, D. W. Lee and K. M. Ok, *Inorg. Chem*., 2014, **53**, 5240−5245.
[S9]	J. V. Caspar, E. M. Kober, B. P. Sullivan and T. J. Meyer, *J. Am.Chem. Soc.*, 1982, **104**, 630−632.
[S10]	J. V. Caspar and T. J. Meyer, *J. Am. Chem. Soc*., 1983, **105**, 5583−5590.
